# Jun Ginger Extract Improves Cold‐Induced Asthma by Inhibiting Airway Inflammation via PI3K/AKT Pathway

**DOI:** 10.1002/fsn3.71249

**Published:** 2025-11-19

**Authors:** Xueqing Wang, Xiaoxiong Song, Haokang Ding, Ziyan Liu, Fengjiao Deng, Hui Yan, Bin Yu

**Affiliations:** ^1^ Jiangsu Collaborative Innovation Center of Chinese Medicinal Resources Industrialization, National and Local Collaborative Engineering Center of Chinese Medicinal Resources Industrialization and Formulae Innovative Medicine Nanjing University of Chinese Medicine Nanjing China; ^2^ Jiangsu Key Laboratory of Efficacy and Safety Evaluation of Traditional Chinese Medicine Nanjing University of Chinese Medicine Nanjing China

**Keywords:** cold‐induced asthma, jun ginger, network pharmacology, Penh, PI3K/Akt pathway

## Abstract

Jun Ginger (JG), a differently processed product from conventional dried ginger (CDG), lacks research on its health benefits. The study was designed to elucidate the bioactive components and therapeutic mechanisms of JG for cold‐induced asthma (CA). In the present study, after the extraction methods of different processing products were optimized, the components of the aqueous‐soluble extract of JG (JGAE) were identified by LC‐TOF‐MS. The protection of JGAE against CA was explored using CA mice and lipopolysaccharide (LPS)‐injured BEAS‐2B cells. After the metabolomics analysis, network pharmacology prediction and molecular docking test were performed, the effect of JGAE in regulating the phosphatidylinositol 3′‐kinase/protein kinase B (PI3K/Akt) pathway was explored and validated by the PI3K inhibitor LY294002 and activator 740Y‐P. The results showed that JGAE outperformed CDG and volatile oil of JG in ameliorating CA, and gingerols were the main components. JGAE decreased the Penh value, inflammatory cells, Th2‐type cytokines and MUC5AC, and increased aquaporin 1 (AQP1) and AQP5. Metabolomics analysis indicated that the acidic metabolites were restored after JGAE treatment. Network pharmacology and molecular docking results suggested that downregulating the PI3K/Akt pathway might be the key mechanism, which was validated by the PI3K inhibitor and activator. These findings indicate that JG is a potential dietary intervention for CA management by downregulating the PI3K/Akt pathway, and gingerols might be the main active component.

## Graphical abstract

Jun Ginger extract improves cold‐induced asthma (CA) by inhibiting airway inflammation via the PI3K/Akt pathway. Specifically, JGAE (aqueous‐soluble extract of Jun ginger) outperformed CDG (conventional dried ginger) in ameliorating CA. JGAE decreased the Penh value, inflammatory cells, Th2‐type cytokines and MUC5AC, and increased aquaporin 1 (AQP1) and AQP5 in CA mice and LPS‐injured BEAS‐2B cells. The involved mechanism was related to suppressing the excessive activation of the PI3K/Akt pathway.

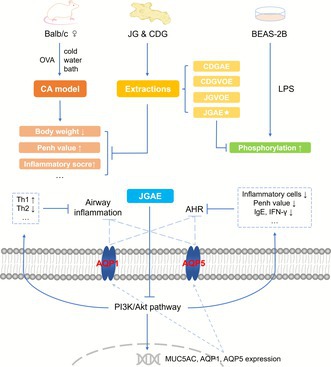

**Abbreviations**
AHR: airway hyperresponsiveness
AQP1: aquaporin 1
AQP5: aquaporin 5
BALF: bronchoalveolar lavage fluid
BEAS‐2B: normal human bronchial epithelial cells
BHR: bronchial hyperresponsiveness
CA: cold‐induced asthma
CCK‐8: cell counting kit 8
CDG: conventional dried ginger
CDGAE: aqueous‐soluble extract of conventional dried ginger
CDGVOE: volatile oil extract of conventional dried ginger
DG: dried ginger
ELISA: enzyme‐linked immunosorbent assay
HE: hematoxylin and eosin
IDA: information‐dependent acquisition
IgE: immunoglobulin E
IHC: immunohistochemistry
IL‐4: interleukin‐4
JG: Jun ginger
JGAE: aqueous‐soluble extract of Jun ginger
JGVOE: volatile oil extract of Jun ginger
LGWWJX: Linggan Wuwei Jiangxin Prescription
LPS: lipopolysaccharide
Mech: methacholine
OD: optical density
OVA: ovalbumin
PBS: phosphate buffered saline
Pehn: enhanced pause
PI3K/Akt: phosphatidylinositol 3′‐kinase/protein kinase B
PPI: protein–protein interaction
RIPA: radio immunoprecipitation assay
Th2: T helper 2 cell

## 1 Introduction

Asthma is a classical respiratory disease affecting both children and adults across the world (Zhang, Ruan, et al. 2024; Zhang, Yang, et al. 2024; Zhang, Du, et al. 2024; Yu et al. 2024). A well‐known feature of asthma is chronic airway inflammation, which causes increased responsiveness to various stimuli and leads to airway stenosis and typical symptoms (Eid et al. 2024). The increased industrialization contributes to the rise of asthma incidence, with prevalence rates ranging from 8% to 23% for children globally (Zhou and Tang 2025). Moreover, increasing evidence suggests that long‐term exposure to low temperatures could worsen and even cause bronchitis and asthma attacks (Deng et al. 2020; Du et al. 2019; Zhou et al. 2023). Consequently, the treatment of asthma, especially cold‐induced asthma (CA), continues to pose significant challenges in clinical practice.

Previous research has proved that asthma, including CA, is a multifaceted respiratory disorder and is featured by airway hyperresponsiveness (AHR), bronchial remodeling, and airway inflammation (Barnes 2016). The cascade of airway inflammation is considered one of the initiating factors of asthma attacks and AHR, and can persistently exacerbate asthma symptoms (Busse 1998). This process is always accompanied by changes in the number of various inflammatory cells, along with elevated levels of IgE and Th2 cytokines (Oppenheimer et al. 2022; Hamzeloo‐Moghadam et al. 2022; Ross et al. 2021). Among these cytokines, IL‐13 induces IgE synthesis and production of pro‐inflammatory cytokines (Diao et al. 2017). IL‐5 is pivotal in promoting eosinophil activation and recruitment, while IL‐4 perpetuates airway inflammation by driving T helper 2 cell (Th2) cell differentiation, enhancing allergen sensitization, inducing AHR, and facilitating macrophage polarization (Walsh et al. 1990; Nakagome and Nagata 2024). MUC5AC, the predominant airway mucin secreted by bronchial goblet cells, participates in type 2/eosinophilic inflammation and AHR (Tajiri et al. 2022). Previous studies have confirmed that aquaporin 1 (AQP1) and AQP5 expressions were positively related to the severity of pulmonary oedema and inflammation, while antiasthmatic treatment could reduce asthma by increasing the levels of AQP1 and AQP5 (Sharif et al. 2022; Ijaz et al. 2021). Thus, targeting airway inflammation and the associated Th2 response offers a promising strategy for CA management.

Ginger is known as the root of *Zingiber officinale* Rosc from the Zingiberaceae family and widely cultivated as a food spice with many bioactivities (Assiry et al. 2023; Nazdar et al. 2024). Jun ginger (JG) is a unique form of dried ginger exclusively produced in the area around Junlian country, Sichuan province, China. Its specific cultivar names also include Tieban Jiang (Iron Plate Ginger), Wuzhi Jiang (Five‐Finger Ginger), and Zhugen Jiang (Bamboo Root Ginger) in the local area. Although JG shares the same origin as CDG, their preparation process is markedly different. The JG is produced by a special steaming technique with water vapor, during which the steam is repeatedly captured and reused before the final drying stage (Huang et al. 2024). This process employs stringent control over technical parameters, including the temperature and duration of steaming and drying. While CDG is prepared only by the air‐dried method. According to the Traditional Chinese Medicine Theory, JG possesses more warming and tonic properties without drying feature compared to CDG produced in other regions. In the early years of the People's Republic of China, JG was recognized as the only export‐grade medicinal dried ginger that met the standards of Japanese Kampo medicine. Currently, JG is recognized as a National Geographical Indication Product of Sichuan Province, China.

Despite its potential benefits, the research of JG on the chemical composition and health benefits is still lacking. The PI3K/Akt pathway is generally regarded as the key mechanism of CA in regulating the inflammatory reaction and Th2 cytokines. However, whether the alleviation of JG against CA is related to the PI3K/Akt pathway is also unclear. Presently, the effect of JG and CDG extracts against CA was compared with each other, and the main active components of JG were also identified. Subsequently, the underlying mechanism was explored, including metabolomics and network pharmacology methods. The result was then validated using the specific activator and inhibitor of the potential target.

## 2 Materials and Methods

### 2.1 Materials

CDG and JG were provided by Fumin Agricultural Products Investment Co. Ltd. (Leshan, China). Morphologically, the two DGs exhibit distinct differences. The cross‐section of CDG is pale yellow or light brown with fibrous structures inside, whereas the cross‐section of JG appears dark brown or black with a glassy, nearly translucent texture, as illustrated in Figure S1.

### 2.2 Establishment of Mouse CA Model Induced by Allergic Airway Inflammation Combining Cold Stimulation

Female healthy BALB/c mice (18 to 22 g) of a specific pathogen‐free degree were bought from GemPharmatech Co. Ltd. (Nanjing, China) with the production license of SCXK (Su) 2018–0008. They were kept in the experimental animal center of Nanjing University of Chinese Medicine freely to food and water. The environmental temperature was 26°C, and the humidity was 55%. All operations in the animal experiments were approved by the Animal Ethics Committee of Nanjing University of Chinese Medicine (No. 202202A040, 202207A054 and 202302A058) and comply with the ARRIVE guidelines.

After 1 week of adaptive feeding, the mice were randomly assigned to the control group and the model group. A modified CA model was developed according to the previous report (Tao et al. 2023). Briefly, mice were intraperitoneally injected with 0.2 mL of phosphate buffered saline (PBS) solution containing 20 μg ovalbumin (OVA, Sigma‐Aldrich, St Louis, USA) and 1 mg aluminum hydroxide (Zhhcbio, Shanxi, China) on days 0, 7, and 14. From days 15 to 29, the mice were exposed to 3% aerosolized OVA daily for 30 min with aerosol flow controlled at 0.5 mL/min. Then, the mice were immediately placed in a cold‐water bath for 10 min. The control group was only injected with PBS and sensitized with PBS for aerosolization, while room‐temperature water was used for gavage. The behavior and body weight were monitored throughout the experiment.

### 2.3 Preparations of JGAE, CDGAE, JGVOE and CDGVOE

To prepare the aqueous‐soluble extract of JG (JGAE) and CDG (CDGAE), 108 g of CDG or JG was soaked for 0.5 h and then decocted twice with eight times distilled water for 0.5 h each time. After the extracts were combined, ethanol was added to the extract to achieve a 50% (v/v) concentration. After standing overnight, the mixture was filtered, concentrated, and purified via solid phase extraction. The product was stored at −20°C.

To prepare the volatile oil extract of JG (JGVOE) and CDG (CDGVOE), 108 g of CDG or JG powder was placed into a round‐bottom flask and the steam distillation was performed. After the process was completed, the distillate was cooled and settled to collect the JGVOE and CDGOE by carefully removing excess water from the flask. The products were stored in a dark environment at −20°C.

### 2.4 Mice Grouping and Treating

#### 2.4.1 Constructing the Mouse CA Model

The mice were randomly divided into the control group and the model group. Both groups contained eight mice. The detection sequence of the samples was random and double‐blind.

#### 2.4.2 Comparing the Effects of JGAE, CDGAE, JGVOE and CDGVOE Against CA

The mice were randomly divided into the groups of control, model, JGAE, CDGAE, JGVOE, and CDGVOE according to the random number method. Guilong Kechuanning capsules (GLKCC) were purchased from Guilong Pharmaceutical Co. Ltd. and chosen as the positive control. The CA model was constructed according to the above method. For comparability, the doses were all standardized to 3.6 g/kg, which corresponded to twice the human equivalent dose. Drug administration began on the first day of the OVA challenge and continued for 15 days. Both the control and model groups were administered the same dose of distilled water. Each group contained eight mice.

#### 2.4.3 JGAE Against CA in a Dosage‐Dependent Manner

In this section, the mice were randomly assigned to six groups, including the control, model, JGAE‐low, JGAE‐middle, JGAE‐high and GLKCC groups. According to the principle of equivalent body surface area, the dose for mice is 12 times the clinical human dose. Considering that the common dosage of JG was between 8 g/day and 10 g/day in the clinic, the dosages of JGAE in the low‐dosage group, middle‐dosage group and high‐dosage group were 0.9 g/kg, 1.8 g/kg and 3.6 g/kg. Similarly, the dosage of GLKCC was 3.6 g/kg. Each group contained eight mice.

### 2.5 Chemical Identification of JGAE

The chemical constituents of JGAE were characterized by liquid chromatography–mass spectrometry using a Shimadzu UFLC XR system (Kyoto, Japan) coupled with a Triple TOF 5600 mass spectrometer (SCIEX, USA) equipped with an electrospray ionization (ESI) source. Chromatographic separation was performed on a Shim‐pack XR‐ODS C18 column (100 × 2.0 mm, 2.2 μm) maintained at appropriate temperature. The mobile phase included 0.1% formic acid in water (A) and 0.1% formic acid in acetonitrile (B), with a gradient elution program as follows: 95% A to 70% A (0–5 min), 70% to 30% A (5–11 min), 30% to 0% A (11–17 min), held at 0% A (17–18 min), returned to 95% A (18–20 min), and re‐equilibrated at 95% A (20–21 min). The flow rate was 0.4 mL/min and the injection volume was 5 μL. Mass spectrometric detection was carried out in both positive and negative ESI modes. The mass range was set to m/z 50–1500. Key MS parameters included: ion spray voltage, ±5500 V (positive/negative mode); source temperature, 550°C; ion source gas (GS1 and GS2), 55 psi; declustering potential, 100 V; collision energy, 10 eV. For MS/MS acquisition, an information‐dependent acquisition (IDA) method was employed, selecting the ten most intense ions per cycle with a collision energy of 40 ± 20 eV. Automatic mass calibration was performed using the integrated calibrant delivery system.

### 2.6 Pulmonary Function Tests

Lung function was assessed using body plethysmography (EMKA Technologies, Paris, France). 24 h after the final nebulization, lung function was measured. Methacholine (Mech) was prepared in normal saline at concentrations of 0, 5, 10, 20, and 30 mg/mL. Each mouse was placed in the observation chamber, and the corresponding concentration of Mech was nebulized using the top nebulizer for gradient stimulation. Each concentration of Mech was nebulized for 1 min, and the enhanced pause (Pehn) was recorded within 5 min after each nebulization to evaluate the degree of AHR.

### 2.7 Differential Count of Inflammatory Cells in Bronchoalveolar Lavage Fluid (BALF)

After the mice were anesthetized by sodium pentobarbital (ip, 50 mg/kg), the right lung was ligated. After an endotracheal tube was inserted, the left lung was slowly flushed three times with 0.3 mL of normal saline. The flushing fluid was collected and centrifuged, and the supernatant was obtained. Then, 100 μL of PBS was added to the remaining sediment, and the mixture was gently vortexed. A 20 μL solution was used for cell counting. The remaining cell suspension was used to prepare a cell smear. Briefly, 10 μL of the suspension was placed on one end of a glass slide and spread evenly using another smooth glass slide. After the smear had dried, 200 μL of Wright‐Giemsa staining solution (Beyotime, Shanghai, China) was added. After being washed and dried, the cells were examined under a microscope to be classified and counted.

The counts of the inflammatory cells were performed by their morphological characteristics (Kehoe et al. 2020). (1) Neutrophils: 12–15 μm in diameter with pale pink cytoplasm containing fine lilac granules, and nucleus segmented with coarsely clumped chromatin; (2) Eosinophils: 12–18 μm in diameter with bright orange‐red cytoplasmic granules, nucleus typically bilobed with coarse chromatin; (3) Lymphocytes: 7–16 μm in diameter; nucleus round or slightly indented with densely clumped chromatin, scant sky‐blue cytoplasm; (4) Total inflammatory cells: total nucleated cells.

### 2.8 Pathological Examination of Lung Tissues

Lung tissues were collected immediately after euthanasia by sodium pentobarbital (ip, 150 mg/kg), rinsed with pre‐cooled saline, and fixed in paraformaldehyde. Following dehydration, the tissue was embedded in paraffin and cut into 4‐μm slices. Histological analysis was performed using hematoxylin and eosin (HE) staining. The slices were observed under an upright optical microscope (Olympus CX 41, Japan), and the corresponding inflammation scores were calculated. The infiltration degree of the inflammatory cell was evaluated on a scale from 0 (no inflammation) to 4 (extensive inflammation).

The expressions of MUC5AC, AQP1 and AQP5 in the lung tissues were assessed by the immunohistochemistry (IHC) method with the antibodies against MUC5AC (1:2000, AiFang Biological, Changsha, China), AQP1 (1:6000, Proteintech, Rosemont, USA), and AQP5 (1:5000, Proteintech, Rosemont, USA). In the histological analysis, the positive cells were dyed brown.

### 2.9 Cell Culture and Establishment of LPS‐Induced Cell Inflammation Injury Model

Normal human bronchial epithelial (BEAS‐2B) cells were provided by Beina Chuanglian Biotech Institute (Beijing, China) and cultured in DMEM medium with 10% fetal bovine serum. Cell proliferation was assessed using Cell Counting Kit 8 (CCK‐8, BEB Laboratories Co. LTD., Nanjing, China) to optimize the concentration of LPS. Briefly, BEAS‐2B cells were seeded into 96‐well plates at a density of 8 × 10^3^ cells per well and divided into control and LPS (0.1, 1, 10, 50, 100 μg/mL) groups. After cell adhesion, the corresponding concentrations of LPS were added, and the cells were incubated for 24 h. Then the optical density (OD) value was measured at 450 nm using a microplate reader according to the instructions of the kit.

### 2.10 Optimization of Drug Dosages

The normal BEAS‐2B cells were seeded into 96‐well plates at a density of 8 × 10^3^ per well and divided into the control group and JGAE (1, 5, 25, 50, 100, and 200 μg/mL) groups. CCK‐8 assay was used to evaluate the potential toxic effect of JGAE.

Then, the dosages of JGAE for treating LPS‐injured BEAS‐2B cells were optimized using the CCK‐8 assay as well. Briefly, BEAS‐2B cells were seeded into 96‐well plates and divided into the control group, model group, and JGAE (1, 5, 25, 50, and 100 μg/mL) groups. All groups except the control group were exposed to 10 μg/mL LPS for 24 h, and the JGAE groups were treated with the corresponding concentrations of JGAE. Subsequently, the CCK‐8 assay was employed to optimize the concentration of JGAE.

### 2.11 Enzyme‐Linked Immunosorbent Assay (ELISA) Assays for IL‐4, IL‐5, IL‐13, lgE and IFN‐γ

ELISA kits (Mlbio, Shanghai, China) were used to measure the concentrations of IL‐4, IL‐5 and IL‐13 in the collected BALF and cell culture medium supernatant. Additionally, the contents of IgE and IFN‐γ were measured in the serum of mice. The assay was performed according to the kit instructions. Specifically, Samples, standards, and sample diluent (100 μL/well) were dispensed into their designated wells, followed by the addition of 50 μL/well biotin‐conjugated antibody to all wells. The plate was sealed and incubated for 2 h at room temperature in the dark. After washing with the wash buffer and removing the water on the plate, 100 μL/well of diluted streptavidin‐HRP was added, and the plate was sealed and incubated for 1 h under light‐protected conditions. Following an identical wash cycle, 100 μL/well of TMB substrate solution was introduced before a final 30‐min incubation. The reaction was terminated with 50 μL/well of stop solution, and the absorbance was measured immediately at 450 nm.

### 2.12 Western Blot Analysis

The lung tissues or cells were lysed using radioimmunoprecipitation assay (RIPA) buffer, and the supernatant was collected. After the protein concentration was determined, the samples were mixed with 5× loading buffer and denatured by heating at 95°C for 10 min. The samples with equal protein concentrations were separated by SDS‐PAGE and transferred to PVDF membranes. After the membranes were blocked, they were incubated with the primary antibodies, including AQP1 (1:10000, Proteintech, Rosemont, USA), AQP5 (1:1000, Proteintech, Rosemont, USA), PI3K (1:5000, Abclonal, Wuhan, China), p‐PI3K (1:1000, Abclonal, Wuhan, China), AKT (1:10000, Abcam, Cambridge, UK), p‐AKT (1:1000, Abclonal, Wuhan, China), and β‐actin (1:8000, Bioworld, Bloomington, USA). Then, they were incubated with the secondary antibody (1:8000, Proteintech, Rosemont, USA). The expressions of the above proteins were detected using an image system (LAS4000 mini, GE Healthcare Life Sciences, Piscataway, NJ, USA).

### 2.13 RT‐qPCR Assay

Total RNA was extracted using Trizol reagent (ThermoFisher, CA, USA). Then the RNA content was tested using a NanoDrop One/OneC Microvolume UV–Vis Spectrophotometer (ThermoFisher, CA, USA). cDNA was synthesized via HiScript III RT SuperMix for qPCR (Vazyme, Nanjing, China). Quantification of cDNA was performed on a LightCycler96 fluorescent quantitative PCR instrument (Roche, Basel, Switzerland) using Taq Pro Universal SYBR qPCR Master Mix (Vazyme, Nanjing, China). All PCR primer sequences (listed in Table S1) were designed and provided by Shanghai Sangon Biotech Co. Ltd. (Shanghai, China). The cycle was performed as follows: pre‐denaturation at 95°C for 30 s, followed by 40 cycles of 95°C for 10 s and 60°C for 30 s. Relative gene expression was analyzed using the 2^−ΔΔCt^ method.

### 2.14 Metabolomic Analysis

Lung tissue samples were collected from the mice. After 10 mL of the prepared internal standard and 800 μL of precipitant were added to each sample, the samples were ground for 5 min and then placed at −20°C for 2 h. After centrifugation, 600 μL of the supernatant was transferred to a new EP tube, frozen, and dried. An equal volume of 50% methanol was then added to the samples to dissolve them. The supernatant was obtained after another centrifugation. Metabolites were then detected using a Waters 2777C UPLC (Waters, USA) coupled with a Q Exactive HF Orbitrap Mass Spectrometer (Thermo Fisher, USA). The data was analyzed via Dr. Tom Multi‐Omics Data Mining System (https://biosys.bgi.com/) and visualized via SRplot System (https://www.bioinformatics.com.cn/).

### 2.15 Network Pharmacology and Molecular Docking Analysis

The chemical components of JGAE were collected from the result of the above LC‐TOF‐MS analysis. Their targets were searched using the databases of TCMSP, SwissTargetPrediction and BATMAN‐TCM. Disease‐related targets were screened using the OMIM and GeneCards databases via the keyword “cold asthma”. After deduplication, the CA‐related targets were obtained. A Venn diagram was created to identify the common targets between JGAE and CA. The network between the potential active components and their targets was constructed using Cytoscape 3.7.2. Protein–protein interaction (PPI) networks were constructed using the STRING platform. GO enrichment and KEGG enrichment were conducted to elucidate the potential mechanism of JG against CA. Autodock Vina and AutoDock Tools were used to conduct the molecular docking. −5.0 to −7.0 kcal/mol of binding energy produces stable affinity, and less than −7.0 kcal/mol is a strong affinity. PyMOL software was employed to visualize the docking mode.

### 2.16 Statistical Analysis

All quantitative data were presented as the mean ± SD and analyzed using one‐way ANOVA and Tukey's post hoc test for multigroup comparisons or *t*‐test for two‐group comparisons. GraphPad Prism 8.0 was utilized for data processing and graphing. Statistical significance was defined when *p* value was less than 0.05.

## 3 Results

### 3.1 Construction of Mouse CA Model

After the construction of the CA model, the mice in the control group exhibited smooth fur, normal activity and respiration. In contrast, the mice in the model group displayed notable lethargy, dull fur and pale lips following the atomization stimulations. These symptoms were accompanied by decreased food intake, weight loss, increased nose scratching, sneezing, shortness of breath and wheezing sounds. As illustrated in Figure S2A, the weight gain of mice in the model group was slower. Compared to the control group, the model group showed obvious reductions in body weight after 23 and 30 days of stimulation (*p* < 0.01 and 0.001). The respiratory function of CA patients is notably marked by bronchial hyperresponsiveness (BHR), which can be quantified by the Penh value (Tao et al. 2023). As depicted in Figure S2B, the Penh values of the CA group increased sharply with the increased concentrations of Mech from day 5 to day 30 (*p* < 0.001), indicating that the airways of the model mice exhibited BHR. Additionally, according to the result of the HE stain, the lung tissue morphology of the control mice displayed neatly arranged bronchial wall cells, whereas those of the model mice exhibited notable pathological changes, such as bronchial wall thickening, extensive infiltration of inflammatory cells and luminal narrowing, as shown in Figure S2C. Compared with the control group, the histological scores of the model mice were significantly increased (*p* < 0.001), as shown in Figure S2D. These results indicated that the CA model was successfully constructed in mice.

### 3.2 JGAE Exhibited the Best Protection Against CA

As shown in Figure S3A,B, the body weight in the model group significantly decreased on day 28 in comparison to the control group (*p* < 0.001). JGAE increased the body weight of mice (*p* < 0.05), whereas other groups showed no significant improvement (*p* > 0.05). As illustrated in Figure S3C–F, the Penh values of mice in the model group significantly increased after administration with 10, 20, and 30 mg/mL Mech compared to the control group (*p* < 0.001). At Mech concentrations of 10 and 30 mg/mL, the Penh values in all the treated groups were significantly reduced (*p* < 0.05, 0.01, and 0.001). Moreover, the Penh values also decreased in CDGAE (*p* < 0.01) and JGAE groups (*p* < 0.001) with the stimulation of 20 mg/mL of Mech. Surprisingly, JGAE produced better therapeutic effects than the CDGAE group on the improvements of the body weights on day 28, the Penh values of the mice stimulated by 30 mg/mL of Mech (*p* < 0.05, 0.01). As shown in Figure S3G,H, HE staining results exhibited extensive infiltration of inflammatory cells in the peribronchial area, marked wall thickening, lumen narrowing and increased inflammation score of the lung tissue in the model group (*p* < 0.001). CDGAE, JGAE, and GLKCC significantly alleviated these pathological changes, substantially reducing inflammation scores (*p* < 0.001).

As demonstrated in Figure S4A–D, the total numbers of inflammatory cells, eosinophils, neutrophils and lymphocytes in the BALF of model mice were significantly increased in comparison to the control group (*p* < 0.001). CDGAE significantly reduced the total inflammatory cells, eosinophils and neutrophils (*p* < 0.05 and 0.01), while JGAE and GLKCC reduced all four types of inflammatory cells (*p* < 0.05, 0.01, and 0.001). As shown in Figure S4E–G, the concentrations of Th2‐type cytokines, including IL‐4, IL‐5 and IL‐13, in the BALF of model mice were significantly elevated (*p* < 0.001). CDGAE reduced the secretions of IL‐5 and IL‐13 (*p* < 0.001), while JGAE and GLKCC decreased all three Th2‐type cytokines (*p* < 0.05, 0.01, and 0.001). Interestingly, the JGAE group could further reduce the level of IL‐13 compared to the CDGAE group (*p* < 0.05). As shown in Figure S4H–I, the level of IFN‐γ was reduced and that of IgE was elevated in the serum of the model mice in comparison to the control group (*p* < 0.001). CDGAE, JGAE and GLKCC increased IFN‐γ and decreased IgE significantly (*p* < 0.01 and 0.001). However, CDGVOE only reduced IgE levels (*p* < 0.01).

The above results indicated that JGAE produced the most satisfactory therapy against CA in comparison to the other extracts. Specifically, it significantly reduced the Penh value, decreased the number of inflammatory cells and the secretions of Th2 cytokines in BALF, and alleviated inflammatory cell infiltration in the lung tissue of CA mice. Additionally, JGAE effectively reduced IFN‐γ, increased IgE in serum, and improved inflammation scores. Therefore, its potential active components and underlying mechanisms against CA were explored in the following research.

### 3.3 The Chemical Profile of JGAE

Eleven compounds in positive ion mode and six compounds in negative ion mode were identified as the main ingredients according to their intensities, as illustrated in Table 1. The extract iron chromatography and MS/MS spectrum of JGAE in positive/negative ion modes are exhibited in Figures S5 and S6. After removing duplicates, 14 main compounds were obtained. Furthermore, the top ten abundant components were identified as 6‐Shogaol, 6‐Gingerol, Gingerenone A, 1‐Dehydro‐6‐gingerdione, α‐Zingiberene, 10‐Shogaol, Acetoxy‐6‐gingerol, 8‐Gingerdione, 6‐Paradol and Citral. Notably, the gingerol components (such as 6‐shogaol, 6‐gingerol, and 10‐shogaol) were abundant in JGAE.

**TABLE 1:** Identification of the main chemical components of JGAE with LC‐TOF‐MS.

No.	T/min	Compound	Formula	Mode	Intensity	MS^1^	MS^2^	ppm
1	9.653	Citral	C_10_H_16_O	[M + H]^+^	19,145	153.1272	41.0481, 60.9911, 81.0733, 91.0578, 97.0678	0.4
2	11.951	Zingiberone A	C_21_H_24_O_5_	[M + H]^+^	86,963	357.1693	131.0502,137.0612, 145.0659, 177.0912	0.9
3	12.368	4‐Gingerol	C_15_H_20_O_3_	[M + H]^+^	8264	249.1483	177.0919, 145.0663, 117.0724	0.8
4	13.891	5‐Deoxy‐6‐Gingerol	C_17_H_26_O_3_	[M + H]^+^	21,954	279.1954	137.0601, 103.0567	0.2
5	14.056	α‐Zingiberene	C_15_H_24_	[M + H]^+^	52,172	205.1951	123.1181, 93.0728, 81.0740, 79.0585, 107.0875, 41.0452, 135.1178, 149.1340, 55.0606, 67.0586	0.2
6	14.272	6‐Gingerol	C_17_H_24_O_3_	[M + H]^+^	410,311	277.1798	137.0605, 145.0656, 177.0909, 117.0718	1.2
7	15.011	6‐Gingerdione	C_17_H_24_O_4_	[M + H]^+^	15,304	293.175	137.0615, 151.0762, 167.1081, 177.0907, 191.1031	0.9
8	17.304	8‐Gingerdione	C_19_H_28_O_4_	[M + H]^+^	25,041	321.2058	137.0611, 163.0761, 131.0506	0.8
9	17.745	1‐Dehydro‐6‐Gingerone	C_17_H_22_O_4_	[M + H]^+^	57,758	291.159	177.0561, 43.0639, 71.0904, 291.1595, 137.0594	0.4
10	18.188	10‐Gingerol	C_21_H_32_O_3_	[M + H]^+^	40,964	333.2425	137.0614, 177.0917, 145.0660, 117.0719, 162.0670	0.3
11	19.774	Acetoxy‐6‐Gingerol	C_19_H_28_O_5_	[M + H]^+^	32,584	338.1415	149.0984, 81.0752, 133.1007, 111.0850	0.2
12	13.643	Zingerone A	C_21_H_24_O_5_	[M−H]^−^	7206	355.1549	83.0517, 219.1032, 355.1521, 121.0322, 204.0836	0.4
13	13.901	Zingerone	C_11_H_14_O_3_	[M−H]^−^	2730	193.0881	193.0857, 177.0584	0.6
14	14.311	6‐Gingerdione	C_17_H_24_O_4_	[M−H]^−^	5435	291.1602	176.0455, 175.0404, 191.0702	0.2
16	14.48	6‐Gingerol	C_17_H_26_O_4_	[M−H]^−^	135,850	293.1767	221.1544, 220.1464, 236.1054, 205.1232	0.8
15	17.433	5‐Deoxy‐6‐Shogaol	C_17_H_26_O_3_	[M−H]^−^	2286	277.1818	233.1908, 277.1792	0.2
17	17.983	4‐Gingerol	C_15_H_20_O_3_	[M−H]^−^	1350	247.1349	247.1337, 133.0288, 203.1458, 89.0409	0.4

### 3.4 The Improvements of Body Weight, BHR and Morphology by JGAE

**FIGURE 1:**
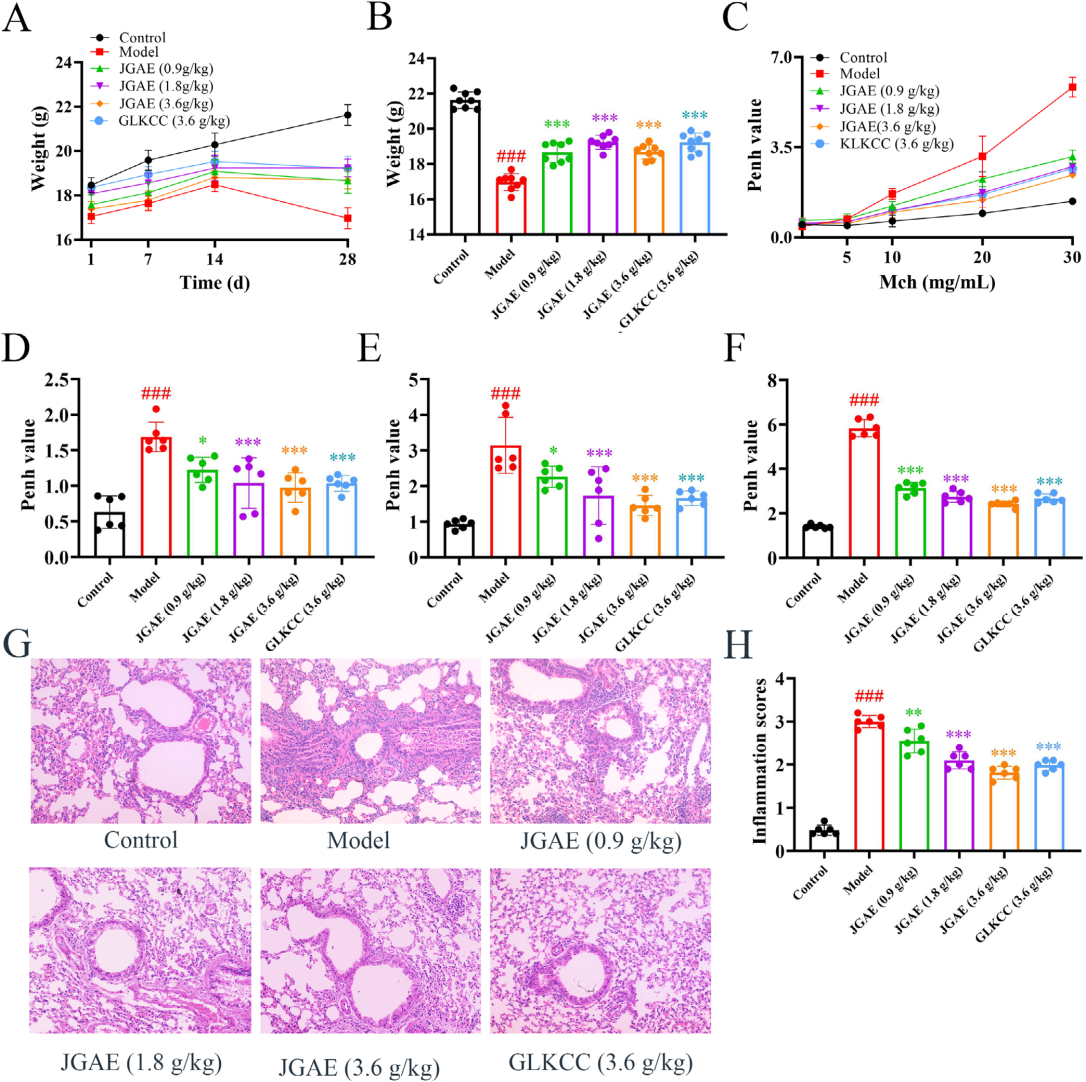
JGAE improved body weight, Penh value and pathological score of the CA mice in a dose‐dependent manner. (A) The body weights of the mice throughout the experiment (*n* = 8). (B) The body weight of the mice on day 28 in each group (*n* = 8). (C) The change trends of the Penh value of the mice along with the increase of the Mech concentration (*n* = 6). (D–F) The Penh values of the mice stimulated by 10, 20 and 30 mg/mL of Mech, respectively (*n* = 6). (G–H) Pathological changes and inflammatory scores of lung tissues in different groups (×200, *n* = 6). *###p* < 0.001 versus the control group; *p < 0.05, **p < 0.01, ***p < 0.001 versus the model group.

Figure 1A,B demonstrated that the three doses of JGAE promoted weight recovery compared to the model group (*p* < 0.001). Figure 1C–F showed that JGAE decreased the Penh value of CA mice to alleviate the BHR in a dose‐dependent manner (*p* < 0.05 and 0.001). Figure 1G,H showed that JGAE dose‐dependently ameliorated the morphology and decreased the inflammation scores of the pulmonary tissue of CA mice (*p* < 0.01 and 0.001). Thus, JGAE exhibited satisfactory protective effects on weight recovery, BHR and histopathological alterations in CA mice.

### 3.5 Dose‐Dependent Anti‐Inflammatory Effects of JGAE in CA Mice

Asthma is featured by bronchial inflammation, which involves activation of various immune cells and release of numerous cytokines, such as IL‐4, IL‐5 and IL‐13 (Zhang, Yang, et al. 2024; Jin et al. 2019). As shown in Figure S7A–D, the differential counting result of inflammatory cells in BALF showed that JGAE markedly reduced the total inflammatory cells, eosinophils, neutrophils, and lymphocytes (*p* < 0.05, 0.01 and 0.001). Figure S7E–I illustrated that JGAE reduced IL‐4, IL‐5 and IL‐13 levels in BALF, and induced an increase of IFN‐γ and a decrease of IgE in serum (*p* < 0.05, 0.01 and 0.001). These results also displayed that JGAE alleviated CA‐induced airway inflammation in a dose‐dependent manner.

### 3.6 Inhibition Effect of JG on Mucus Secretion in CA Mice

The increase of mucus secretion in the airway characterizes CA disorder. MUC5AC, a mucin produced by goblet cells in the tracheobronchial epithelium, is considered the principal component of airway mucus and plays a pivotal role in the development of CA (Kong et al. 2021). Previous studies also showed that AQP1 and AQP5 levels were significantly reduced in OVA‐induced mouse asthma models, which induced increased alveolar fluid viscosity and mucus plug formation (Koyama et al. 1999).

**FIGURE 2:**
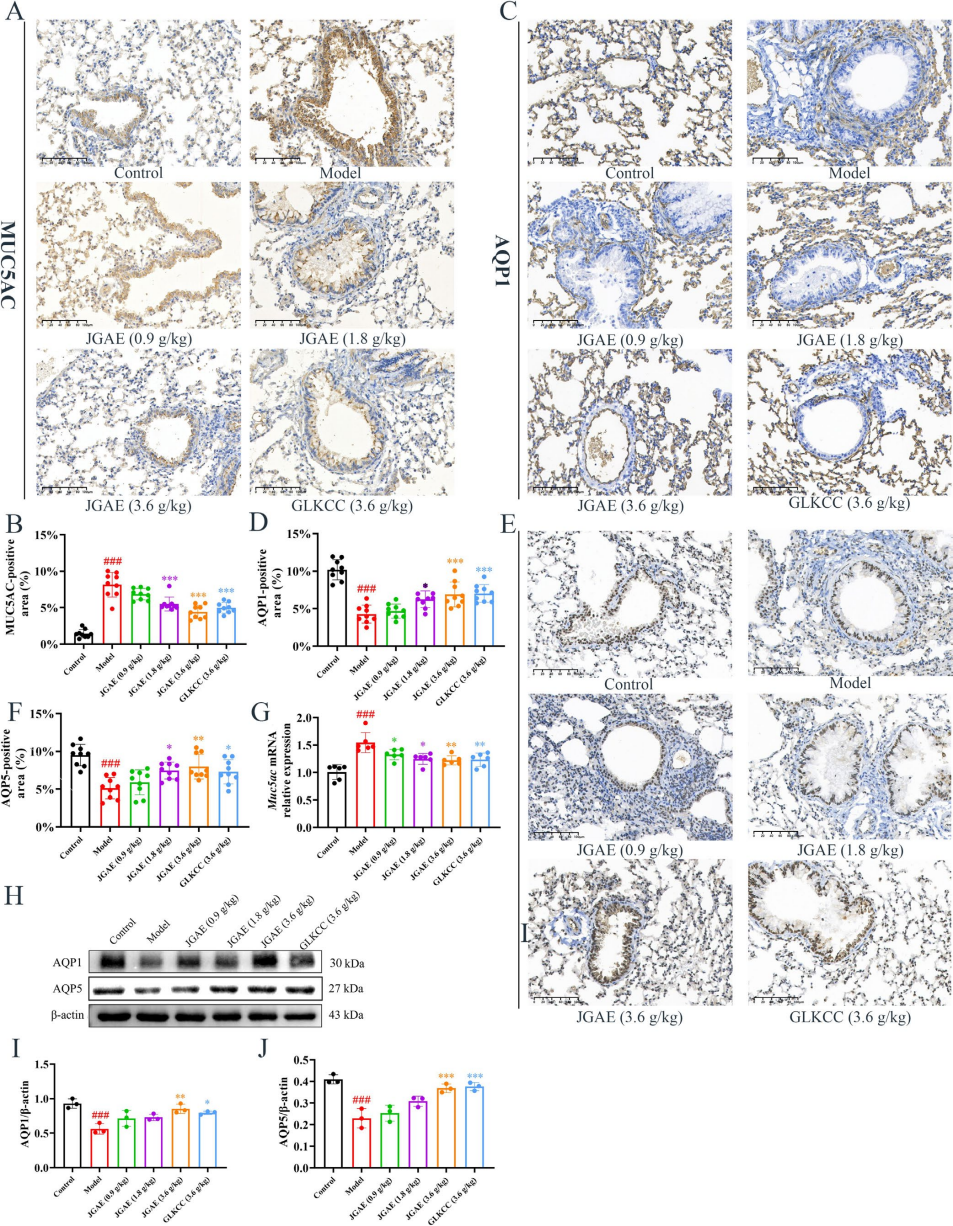
JGAE inhibited the expressions of MUC5AC, AQP1 and AQP5 in the CA mice. (A–F) IHC results of MUC5AC, AQP1 and AQP5 expressions in CA mice with three random visual fields (*n* = 3). (G) The mRNA expression level of *Muc5ac* in CA mice (*n* = 6). (H–J) Representative western blot bands and results of AQP1 and AQP5 expressions in CA mice (*n* = 3). *###p* < 0.001 versus the control group; *p < 0.05, **p < 0.01, ***p < 0.001 versus the model group.

As shown in Figure 2A–F, the immunohistochemistry results showed a notable increase in MUC5AC expression in the lung tissue of CA mice compared to the control group (*p* < 0.001). Conversely, the expressions of AQP1 and AQP5 were markedly reduced (*p* < 0.001). JGAE significantly reduced MUC5AC (*p* < 0.001) and increased AQP1 and AQP5 (*p* < 0.05, 0.01 and 0.001). Figure 2G–J detailed the protein and mRNA expression levels of *Muc5ac*, *Aqp1* and *Aqp5*, and the results were consistent with those of immunohistochemistry. Specifically, the levels of *Aqp1* and *Aqp5* were decreased in the model group, whereas *Muc5ac* was elevated markedly (*p* < 0.001). However, JGAE increased *Aqp1* and *Aqp5*, and decreased *Muc5ac* obviously (*p* < 0.05, 0.01 and 0.001).

### 3.7 Construction of the In Vitro CA Model and Optimization of JGAE Concentration

LPS is widely used to induce the inflammation of BEAS‐2B cells by increasing monocyte chemotactic activity (Shang et al. 2019; Chen and Zheng 2023). In the present study, the viability of BEAS‐2B cells decreased significantly in a dose‐dependent manner after being attacked by LPS between 1.0 and 100 μg/mL as shown in Figure S8A (*p* < 0.05, 0.001). Considering that both excessive and insufficient cellular damage were not conducive to inducing the protection effects of JGAE, 10 μg/mL of LPS was selected to induce the inflammatory model of BEAS‐2B cells in the subsequent in vitro experiments.

Initially, the CCK‐8 assay was adopted to measure the toxic concentration range of JGAE. As shown in Figure S8B, the viability of the normal BEAS‐2B cells was significantly reduced when the concentration of JGAE reached up to 200 μg/mL (*p* < 0.001), indicating the occurrence of cytotoxicity. So, 1, 5, 25, 50 and 100 μg/mL of JGAE were used for its concentration optimization. As illustrated in Figure S8C, 10 μg/mL of LPS markedly reduced the viability of BEAS‐2B cells (*p* < 0.001). JGAE exhibited significant protection on LPS‐injured BEAS‐2B cells at the concentrations of 5, 25, 50, and 100 μg/mL (*p* < 0.05 and 0.001). Therefore, 5, 25 and 50 μg/mL of JGAE were selected in the subsequent experiments.

### 3.8 Dose‐Dependent Inflammatory Inhibition of JGAE In Vitro

**FIGURE 3:**
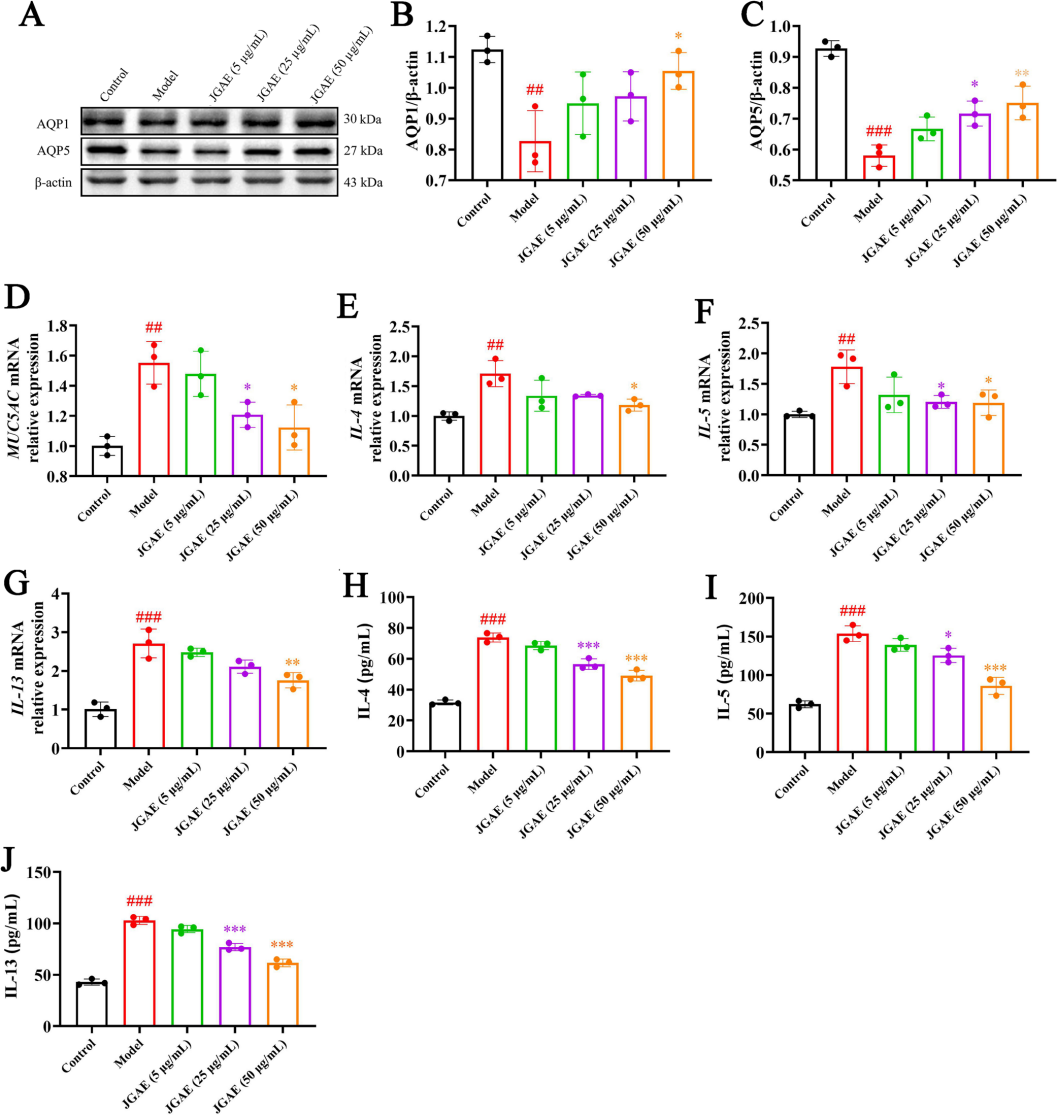
Protective effects of JG in BEAS‐2B cells injured by LPS. (A–C) Representative western blot bands and results of AQP1 and AQP5 expressions (*n* = 3). (D) The mRNA expression level of *MUC5AC*. (E–G) The mRNA expression levels of *IL‐4, IL‐5* and *IL‐13* (*n* = 3). (H–J) The concentrations of IL‐4, IL‐5 and IL‐13 in the culture medium (*n* = 3). ##*p* < 0.01, ###*p* < 0.001 versus the control group; *p < 0.05, **p < 0.01, ***p < 0.001 versus the model group.

As depicted in Figure 3A–C, LPS reduced the expressions of AQP1 and AQP5 significantly (compared to the control group, *p* < 0.01 and 0.001), while JGAE upregulated their expressions (compared to the model group, *p* < 0.05 and 0.01). Moreover, LPS elevated the *MUC5AC* mRNA level compared to the control group (*p* < 0.01), as shown in Figure 3D. Similarly, JGAE also markedly suppressed its expression (*p* < 0.05).

Subsequently, RT‐qPCR and ELISA assays were applied to evaluate the inflammatory reaction in vitro. As illustrated in Figure 3E–G, the gene expression levels of *IL‐4*, *IL‐5* and *IL‐13* were significantly elevated in the model group compared to the control group (*p* < 0.01 and 0.001), which proved that LPS induced an inflammatory reaction in BEAS‐2B cells. However, JGAE significantly reduced the mRNA levels of *IL‐4*, *IL‐5* and *IL‐13* compared to the model group (*p* < 0.05, 0.01). As depicted in Figure 3H–J, the ELISA results further supported these findings and showed significant reductions of IL‐4, IL‐5, and IL‐13 after JGAE administration (*p* < 0.05 and 0.001). Furthermore, JGAE displayed an obvious dose‐dependent inhibition on the above indices.

### 3.9 Metabolomics Analysis Result

The metabolomic analysis was performed on the pulmonary tissues among the groups of control, model and JGAE (3.6 g/kg). The mass spectrometry data were analyzed using the BMDB, mzCloud, and ChemSpider online databases, and a total of 97 metabolites were identified, as shown in Table S2. Based on the criteria of *p* < 0.05 and fold change (FC) > 1.2 or < 0.833, volcano plots were generated to illustrate these results in Figure S9A,B. To further explore the differential metabolites, a heatmap was plotted. In Figure S9C, 29 metabolites, including L‐Threonine, DL‐Alanine, and Proglumide, were significantly upregulated, while 12 metabolites, such as L‐Arginine, DL‐Arginine, and (+)‐Lysergic acid, were significantly downregulated in the model group compared to the control group. It is generally believed that both L‐Threonine and DL‐Alanine participate in the activation of immune function, including inducing the proliferation and secretion of lymphocytes (Rzetecka et al. 2025; Zhao et al. 2015). IL‐4 perpetuates airway inflammation by driving T helper 2 cell (Th2) cell differentiation, IL‐5 is pivotal in promoting eosinophil activation and recruitment, and IL‐13 induces IgE synthesis and production of pro‐inflammatory cytokines (Diao et al. 2017). The increases of L‐Threonine and DL‐Alanine indicated that the activation of lymphocytes, which were consistent with the enhancement of the Th2 cytokines in the present experiment. Moreover, recent research found that L‐Arginine could increase NO and decrease reactive oxygen metabolites levels to inhibit the inflammation reaction of children with asthma (Yamamoto et al. 2024). The reduction of L‐arginine in the model group of the present study indicated the deterioration of respiratory function.

As depicted in Figure S9D, the JGAE significantly upregulated 6 metabolites, including acetohydroxamic acid and 7‐ketocholesterol, and downregulated 9 metabolites, such as L‐aspartic acid and Indoleacetic acid. Meanwhile, many acidic metabolites were restored, including ophthalmic acid, indoleacetic acid and norcholic acid. Soga reported that the content of serum ophthalmate increased 5‐fold after acetaminophen treatment, while the GSH level dropped dramatically. So, serum ophthalmate reflects liver GSH disorder triggered by oxidative stress (Soga et al. 2006). Shuai found that pulmonary hypertension induced an increase of 3‐indoleacetic acid, and considered that it was a promising target for early diagnosis and treatment of the disease (Shuai et al. 2025). Currently, the research on norcholic acid mainly focuses on bile acid metabolism, and more evidence shows that norcholic acid might play a key role in microbial profiles (Tian et al. 2025). However, the research on the relationship between norcholic acid and asthma is still lacking.

Then, a KEGG enrichment was performed according to the differential metabolites between the model group and the JGAE group, and the result is shown in Figure S9E. Previous study revealed that tryptophan metabolism abnormality was related to Hashimoto's thyroiditis, and tryptophan could alleviate the disease by regulating T cell function and the PI3K‐Akt pathway, which suggested tryptophan metabolism might be a potential therapeutic target for Hashimoto's thyroiditis (Zhang et al. 2025). Manna reported that L‐cysteine supplementation reduced NF‐κB phosphorylation and the secretion of TNF‐α, MCP‐1, IL‐8, IL‐1β, and IP‐10 by increasing AMPK phosphorylation and PPARγ expression against vascular inflammation in diabetes mediated by PI3K (Manna and Jain 2013). Ji reported that dietary methionine could enhance the immune and antioxidant capacity of this fish species by inducing the PI3K/Akt/Nrf2 pathway and inhibiting the NF‐κB pathway (Ji et al. 2020). Obviously, the PI3K/AKT pathway might play a crucial role in JGAE against CA.

### 3.10 Network Pharmacology Analysis

**FIGURE 4:**
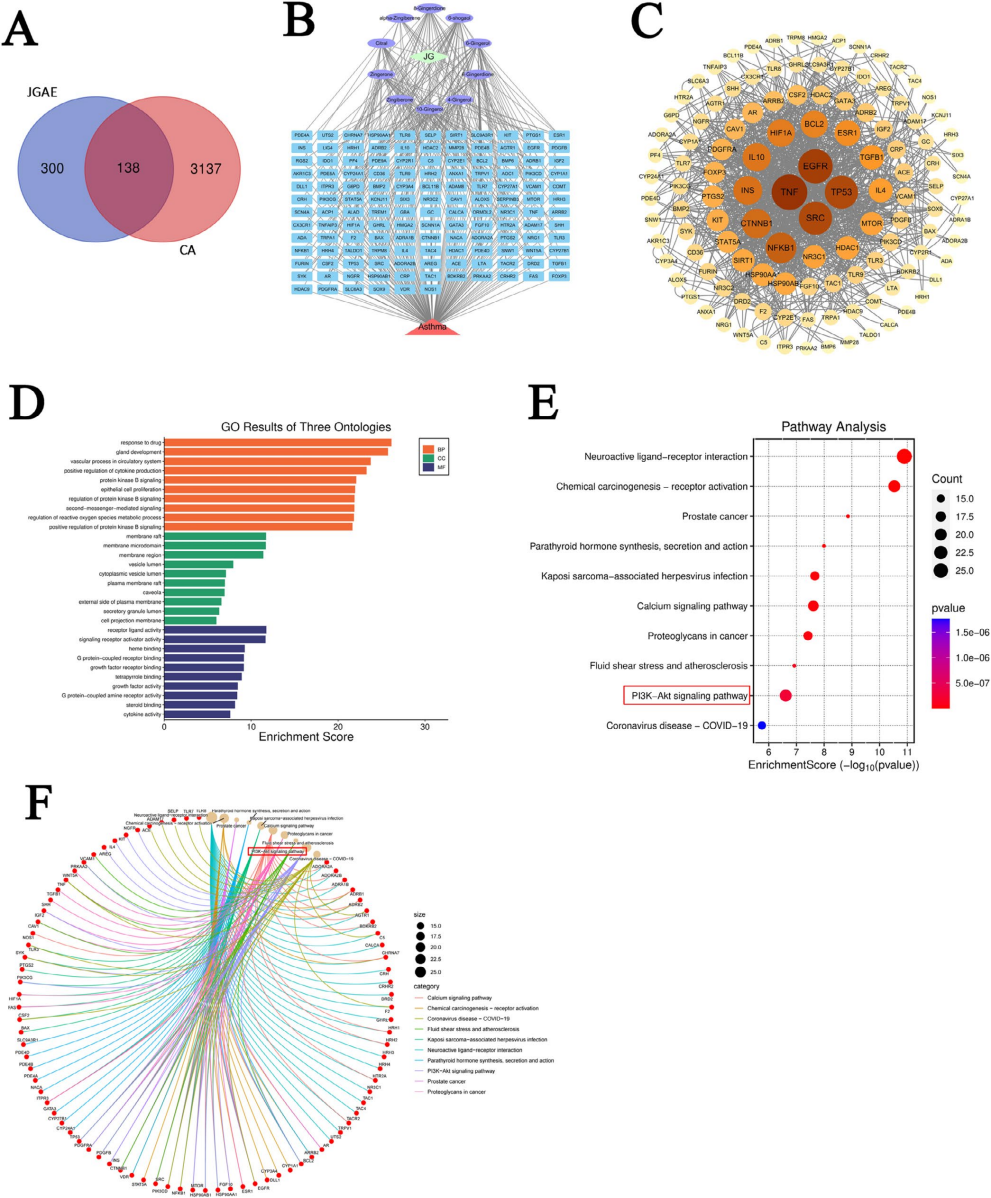
The mechanism of JGAE against CA via network pharmacology analysis. (A) 138 targets were identified by the intersection of JGAE targets and CA targets. (B) Network diagram of JGAE‐components‐targets‐CA. (C) PPI diagram of the common targets, in which darker colors and larger fonts indicate higher degree values. (D) Results of GO enrichment analysis on functional targets. (E) Results of KEGG enrichment analysis on signaling pathways. (F) Chord plot displaying the relationship between the targets and signaling pathways.

The Venn analysis result was shown in Figure 4A, which suggested that there were 138 common targets between CA and the identified components. The network relationship of “JGAE‐components‐targets‐CA” is displayed in Figure 4B. The result of the PPI network diagram for the 138 common targets displayed that TNF, TP53, NFKB1, IL10 and IL4 were the key targets as shown in Figure 4C. According to the result of GO enrichment analysis (Figure 4D), the biological processes affected by JGAE mainly involved positive regulation of cytokine production and protein kinase B signaling, the cellular components mainly involved the membrane microdomain and membrane region, and the molecular functions concerned signaling receptor activator activity and G‐protein‐coupled receptor binding. Figure 4E displayed the KEGG pathway result, which indicated that the regulation of the PI3K/Akt signaling pathway might be the key mechanism of JGAE against CA. Figure 4F further showed the relationship between the targets and signaling pathways, including the PI3K/Akt pathway.

**FIGURE 5:**
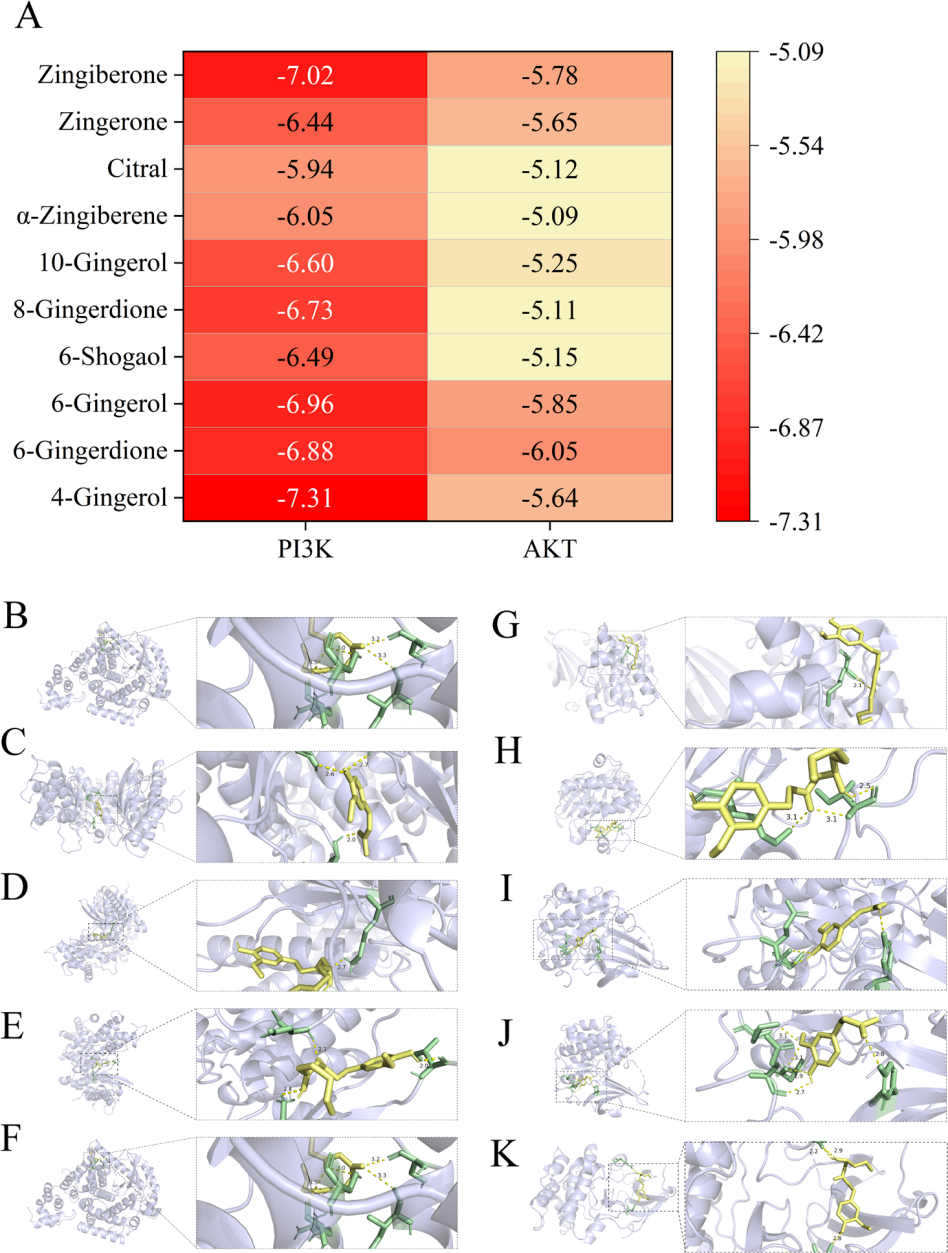
The components in JGAE displayed stable affinity with PI3K and AKT. (A) was binding energies (kcal/mol) between the active ingredients and potential targets. (B–F) were the docking models of PI3K with 4‐gingerol, zingiberone, 6‐gingerol, 6‐gingerdione and 8‐gingerdione, respectively. (G–K) were the docking models of AKT with 6‐gingerdione, 6‐gingerol, zingiberone, zingerone and 4‐gingerol, respectively. The components, targets and amino acid residues were labeled with yellow, gray and green, respectively.

Subsequently, the molecular docking suggested that all of the ten ingredients in JGAE exhibited fairly strong affinities to PI3K and AKT (≤ −5.0 kcal/mol), especially for PI3K, as shown in Figure 5A. Moreover, the gingerols were the main component. Both of the top five binding modes of PI3K and AKT were visualized in Figure 5B–K.

### 3.11 JGAE Ameliorated CA by Downregulating PI3K/Akt Pathway In Vivo and In Vitro

**FIGURE 6:**
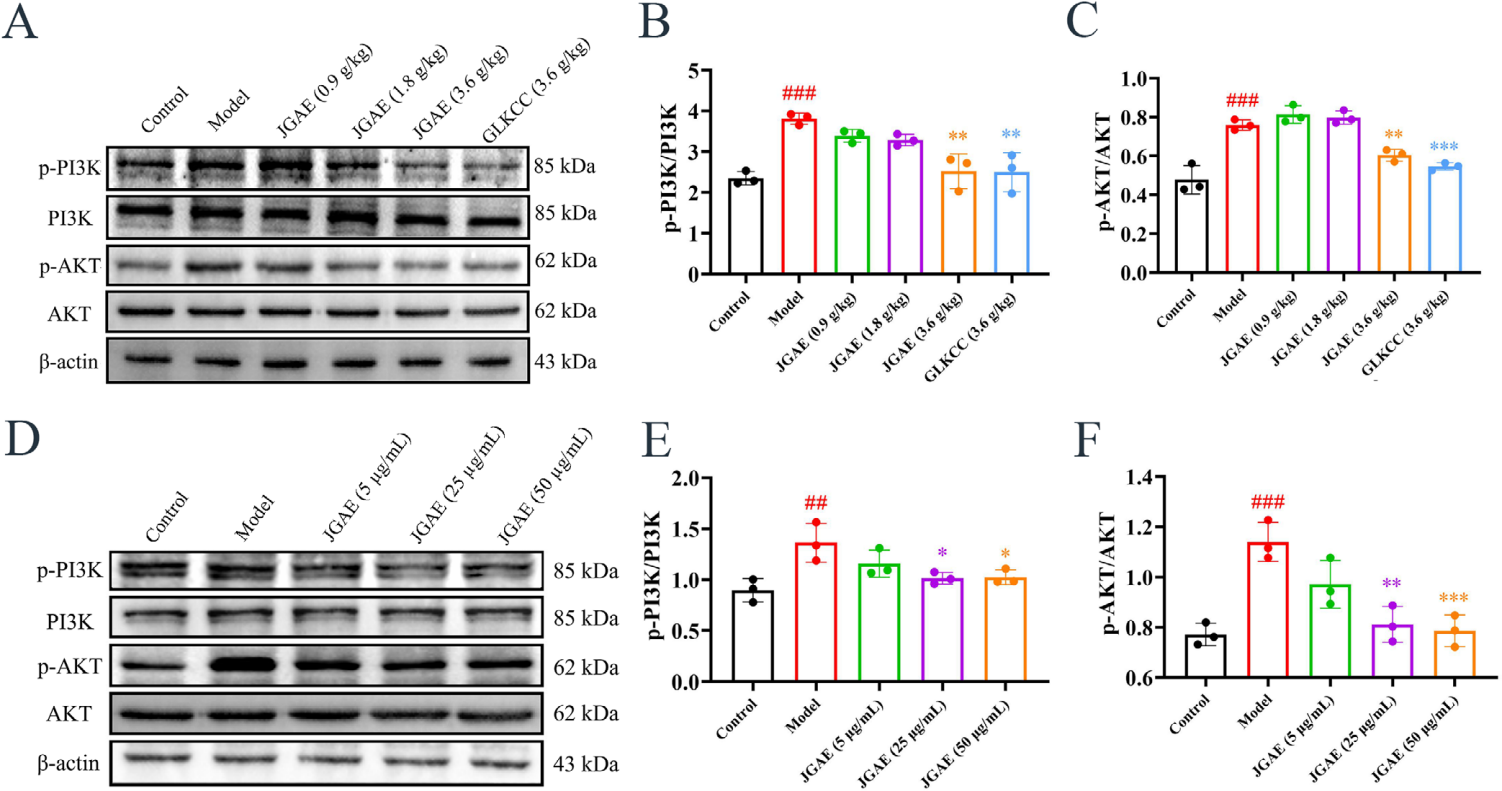
JGAE suppressed PI3K/Akt pathway in CA mice and LPS‐injured BEAS‐2B cells. (A–C) Representative western blot bands and quantitative analysis of p‐PI3K, PI3K, p‐Akt, Akt and β‐Actin in CA mice (*n* = 3). (D–F) Representative western blot bands and quantitative analysis of p‐PI3K, PI3K, p‐Akt, Akt and β‐Actin in BEAS‐2B cells (*n* = 3). ##*p* < 0.01, ###*p* < 0.001 versus the control group; *p < 0.05, **p < 0.01, ***p < 0.001 versus the model group.

To validate the results of the above network pharmacology, the PI3K/Akt signaling pathway was detected via in vivo and in vitro studies. As shown in Figure 6A–C, the ratios of p‐PI3K/PI3K and p‐Akt/Akt in the pulmonary tissue of CA mice were significantly elevated (*p* < 0.001), while JGAE and GLKCC markedly reduced the ratios (*p* < 0.01 and 0.001), which suggested that the mechanism of JGAE ameliorating CA might be involved in inhibiting the PI3K/Akt pathway. Figure 6D–F show the results of BEAS‐2B cells. Similarly, LPS induced a significant increase in p‐PI3K/PI3K and p‐Akt/Akt ratios (*p* < 0.01, 0.001), while JGAE still decreased the ratios markedly (*p* < 0.05, 0.01, and 0.001). These results confirmed that JGAE effectively inhibited PI3K/Akt activation in CA mice and LPS‐injured BEAS‐2B cells, consistent with the result of network pharmacology analysis.

### 3.12 Validation of JGAE Regulating PI3K/Akt Pathway In Vitro

**FIGURE 7:**
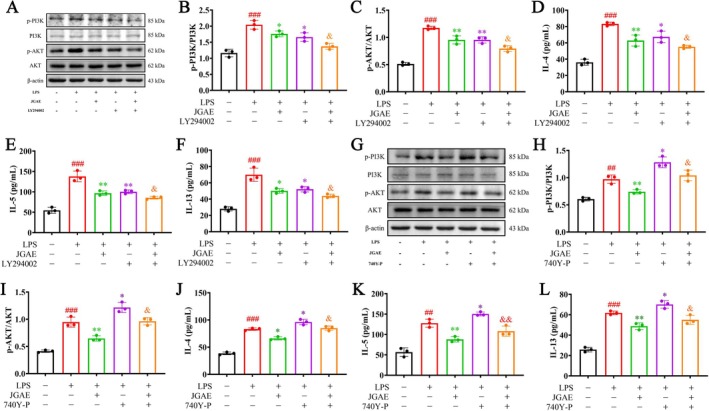
The validation of JGAE against CA via suppressing the PI3K/Akt pathway in vitro. (A–C) Representative western blot bands and quantitative analysis of p‐PI3K, PI3K, p‐Akt, Akt and β‐Actin in different groups after LY294002 treatment (*n* = 3). (D–F) The secretion levels of IL‐4, IL‐5 and IL‐13 after LY294002 treatment (*n* = 3). (G–I) Representative western blot bands and quantitative analysis of p‐PI3K, PI3K, p‐Akt, Akt and β‐Actin in different groups after 740Y‐P treatment (*n* = 3). (J–L) The secretion levels of IL‐4, IL‐5 and IL‐13 after 740Y‐P treatment (*n* = 3). ###*p* < 0.001 versus the control group; *p < 0.05, **p < 0.01, ***p < 0.001 versus the model group.

The PI3K inhibitor LY294002 and activator 740Y‐P were employed to validate the regulation of JGAE on the PI3K/Akt pathway. The result showed that LY294002 inhibited the phosphorylation of PI3K, while 740Y‐P induced its phosphorylation, as previously reported (Gu et al. 2024; Zhang, Du, et al. 2024; Qian et al. 2024; Wang et al. 2021). As depicted in Figure 7A–F, western blot and ELISA results indicated that LPS increased the ratios of p‐PI3K/PI3K, p‐Akt/Akt, and the secretions of IL‐4, IL‐5, and IL‐13 (*p* < 0.01, 0.001). In contrast, JGAE or LY294002 significantly downregulated the PI3K/Akt pathway (*p* < 0.05, 0.01). Moreover, as the downstream of the pathway, the secretions of IL‐4, IL‐5, and IL‐13 were reduced as well compared to the model group (p < 0.05, 0.01). Additionally, JGAE and LY294002 exhibited an obvious synergic effect in regulating the PI3K/Akt pathway and ameliorating the release of the inflammation reaction in the downstream of the pathway (*p* < 0.05). As shown in Figure 7G–L, 740Y‐P activated the PI3K/Akt pathway and further increased IL‐4, IL‐5 and IL‐13 levels compared to the LPS group (*p* < 0.05), which were all counteracted by JGAE (p < 0.05, 0.01). These findings validated that the mechanism of JGAE against CA was involved in downregulating the PI3K/Akt pathway.

## 4 Discussion

Asthma is regarded as a significant public health challenge throughout the world, affecting approximately 10% of the adult population and imposing considerable social and economic burdens (Hyrkäs‐Palmu et al. 2022). More and more evidence indicates that excessively low ambient temperature significantly elevates asthma‐related hospitalizations, exacerbates respiratory symptoms, impairs lung function, and aggravates airway inflammation (Deng et al. 2020; Hyrkäs‐Palmu et al. 2022; Han et al. 2023). Moreover, Low temperatures may exacerbate asthma by triggering BHR and disrupting the integrity of the epithelial barrier of the respiratory tract (Tao et al. 2023). The current treatment for CA primarily relies on glucocorticoids or β‐agonist agents. However, these therapeutic drugs are associated with adverse effects such as immunosuppression, electrolyte imbalance and hypertension. They provide only transient relief and are not suitable for long‐term application, and the symptoms and inflammation often relapse upon cessation of therapy (Barnes 2013). These limitations significantly hinder the long‐term improvement of respiratory function in CA patients.

Multiple studies have demonstrated that many folk foods can effectively alleviate asthma symptoms with minimal toxicity and side effects, and they are even suitable for long‐term administration. Ginger has served as both medicine and food for over 5000 years in China and India. Presently, researchers have conducted extensive research on CDG or fresh ginger, including their improvement on asthma, chronic bronchitis and emphysema (Kardan et al. 2019), while the report on JG is lacking. JG is derived from ginger, but its preparation process differs from that of CDG. The former involves repeatedly using collected steam for steaming, while the latter is simply prepared through conventional air drying. This unique processing provides JG with less dry properties according to the Traditional Chinese Medicine principle (Huang et al. 2024). The present study suggested that JG outperformed CDG in ameliorating CA disorder, and JGAE displayed better therapeutic effects than JGVOE, including normalization of airway morphology, alleviation of AHR, inhibition of inflammatory infiltration and the reduction of secretion of the inflammatory cytokines. Obviously, JGAE displayed the best effect on the CA management, and it was selected for further exploration on the potential material basis and mechanism against CA.

The components of JGAE were unknown before the present study. In this experiment, we found that the top 10 ingredients in the JGAE were citral, zingiberone A, 4‐gingerol, 5‐deoxy‐6‐gingerol, α‐zingiberene, 6‐gingerol, 6‐gingerdione, 8‐gingerdione, 1‐dehydro‐6‐gingerone and 10‐gingerol. Obviously, gingerol compounds were highly abundant in JG, and the subsequent results of network pharmacology and molecular docking analysis further confirmed that 10‐gingerol, 4‐gingerol, and 6‐gingerol are likely the main active ingredients responsible for its anti‐CA effects. Moreover, the previous study confirmed that the top 10 abundant ingredients in the mature ginger were 6‐gingerdiol, 6‐gingerol, methyl 6‐gingerol, 5‐acetoxy‐6‐gingerdiol, diacetoxy‐4‐gingerdiol, 8‐gingerdiol and 8‐gingerol, acetoxy‐6‐gingerol, methyl 5‐acetoxy‐6‐gingerdiol and 6‐shogaol, respectively (Tanaka et al. 2015). Compared to the mature ginger, the proportion of gingerdiols in JG decreases significantly, while the levels of shogaols and gingerols increase markedly. Moreover, a recent study confirmed that most of the component concentrations increased significantly as the moisture content decreased during the drying process of DG. However, subsequent thermal processing induced decomposition of nonvolatile constituents, including the shogaols and gingerols (Yang et al. 2025), which have been demonstrated to possess a wide range of bioactive properties, including antioxidant, anti‐inflammatory effects (Abdul Rani et al. 2025).

Penh value is widely used in asthma research because there have been studies reporting that Penh value changes along with the pulmonary resistance variation (Bergren 2001). Airway inflammation plays a critical role in the development of asthma (Kang et al. 2013). Studies have shown that airway inflammation is closely related to an imbalance in the Th1/Th2 immune response (He et al. 2020). With the development of asthma, the excessive secretion of Th2 cytokines, such as IL‐4, IL‐5, and IL‐13, is considered the primary driver of immune dysregulation (Tang et al. 2016). Th1 cytokines, such as IFN‐γ, inhibit IgE production and suppress the Th2 immune response by impairing Th2 cell differentiation and reducing the expression of Th2 cytokines (Ma et al. 2021). Therefore, the strategies that reduce the Th2 cytokines and increase the Th1 cytokines may offer effective approaches to asthma alleviation. Furthermore, as a chronic inflammatory disease in the airway, asthma is characterized by activating various immune cells, including mast cells, dendritic cells, neutrophils, macrophages, basophils and eosinophils (Verkman 2007). MUC5AC, the primary mucin secreted by tracheobronchial epithelial goblet cells, is regarded as a critical component of airway mucus and plays a pivotal role in asthma pathogenesis (Tao et al. 2023; Shang et al. 2019). Besides MUC5AC, the aquaporins of AQP5 and AQP1 have also been implicated in the development of asthma (Chen and Zheng 2023). The present experiments demonstrated that JGAE effectively reduced the levels of Th2 cytokines (IL‐4, IL‐5, and IL‐13) in the BALF of CA mice, and decreased the number of inflammatory cells, including eosinophils, neutrophils and lymphocytes. Moreover, JGAE ameliorated the infiltration of inflammatory cells in lung tissue, increased Th1 cytokine IFN‐γ and reduced IgE to alleviate airway inflammation accompanying asthma attacks. The results of the in vitro experiment further demonstrated the effect of JG against CA by inhibiting the inflammatory reaction. The present study indicated that there was a close relationship between airway inflammation and the imbalance of the Th1/Th2 immune response in CA disease. Furthermore, JG exhibited an obvious amelioration against CA by inhibiting the inflammation reaction and mucus secretion, and the potential mechanism is likely related to modulating the imbalance of Th1/Th2 and modulating the expressions of MUC5AC, AQP1 and AQP5. Previous evidence showed that IFN‐γ suppressed Th2 function, such as the expression of MUC5AC, suggesting that IFN‐γ may regulate asthma development (Potaczek et al. 2020). The present study exhibited that JGAE could increase the IFN‐γ along with the reduction of MUC5AC expression, indicating that IFN‐γ might be a key target of JGAE against CA.

More and more evidence indicates that abnormal amino acid metabolism is involved in the occurrence and development of asthma, and these amino acids are regarded as prognostic indicators for the development of asthma. Till now, the identified amino acids involved in asthma include l‐threonine, dl‐alanine (Kelly et al. 2017), arginine (Cottrill et al. 2023), glycine, serine, threonine and aspartic acid (Jie et al. 2023). Improvement in asthma is often accompanied by the normalization of the above‐mentioned amino acid metabolites (Seo et al. 2018). However, there have been no reports on characteristic metabolites specific to CA till now. The present study identified many new metabolites, which might be involved in the development of CA. Specifically, 29 metabolites, including threonine, alanine and proglumide, were upregulated, and 12 metabolites, including arginine and lysergic acid, were downregulated in the model group. Moreover, JGAE upregulated 6 metabolites, including acetohydroxamic acid and 7‐ketocholesterol, and downregulated 9 metabolites, including aspartic acid and indoleacetic acid. The restored acidic metabolites included ophthalmic acid, indoleacetic acid and norcholic acid, which was reported for its anti‐inflammation effect (Zhu, Boucheron, et al. 2021; Zhu, Sun, et al. 2021).

Although the above results suggested that JGAE alleviated CA by inhibiting airway inflammation and excessive mucus secretion, the underlying mechanism was still unclear. Network pharmacology, as a comprehensive approach rooted in bioinformatics and systems biology, facilitates to exploration of the underlying mechanisms of natural product‐derived drugs and foods. As a transformative paradigm in drug or functional food discovery and development, network pharmacology broadens the scope for finding new therapeutic agents by identifying novel pro‐drugs and targets (Li, Bai, et al. 2020; Li, Li, and Ji 2020). Additionally, network pharmacology contributes to optimizing current drugs, suggesting ways to enhance their efficacy and safety profile (Joshi et al. 2025). By investigating underlying disease mechanisms, network pharmacology deepens our understanding of drug actions within biological systems and the therapeutic processes they involve (Li, Bai, et al. 2020; Li, Li, and Ji 2020). Moreover, network pharmacology provides a systematic approach for analyzing potential side effects, improving risk assessment, and characterizing drug targets to achieve more precise interventions and improved outcomes (Vithalkar et al. 2025). Accordingly, the network pharmacology analysis was performed to investigate the mechanisms of JGAE against CA. A total of 138 common targets were screened by Venn analysis, and the key targets, including TNF, TP53, NFKB1, IL10 and IL4, were also obtained via PPI analysis. GO enrichment analysis indicated that cytokine production, protein kinase B signaling, membrane microdomain and G‐protein‐coupled receptor might be involved in the alleviation of CA by JGAE. KEGG analysis showed that the PI3K‐Akt signaling pathway might be the key mechanism of JGAE against CA. The molecular docking results also confirmed that the PI3K/Akt pathway was the potential target of JGAE, and gingerol components might be the main active ingredients, which were consistent with the result of the above chemical profile.

The PI3K‐Akt signaling pathway plays a crucial role in the development of many inflammatory diseases, including asthma (Athari 2019). It mediates type I allergic reactions by promoting the differentiation, aggregation and activation of eosinophils, as well as stimulating B cells to produce IgE. The pathway is also involved in airway remodeling, epithelial fibrosis and mucus hypersecretion, which contribute to tissue damage and BHR (Zhu, Boucheron, et al. 2021; Zhu, Sun, et al. 2021; Lee et al. 2006). Moreover, the activation of the PI3K/Akt pathway promotes the contraction of bronchial smooth muscle, which then leads to airway narrowing, dyspnea and exacerbating asthma severity (Ma et al. 2020). However, the regulation of the PI3K/AKT pathway on the CA model was explored for the first time in the present study.

To further research and validate the mechanism of JGAE against CA by regulating the PI3K/Akt pathway, the ratios of p‐PI3K/PI3K and p‐Akt/Akt, the secretions of Th2 cytokines were all measured with the coadministration of PI3K inhibitor LY294002 or PI3K activator 740Y‐P. Interestingly, there was a synergic effect between JGAE and LY294002 on downregulating the PI3K/AKT pathway and the generation of the inflammatory cytokines as the downstream of the pathway. Furthermore, the downregulation of JGAE on the pathway was concealed by 740Y‐P. The result suggested that inhibiting PI3K/Akt signaling pathway might be the key mechanism of JGAE suppressing airway inflammation and alleviating CA. The PI3K/Akt pathway, as a classical cascade reaction mechanism, is involved in the onset and progression of many respiratory diseases. More and more Traditional Chinese Medicine extracts are found to possess the potential therapeutic effect against the disorder via PI3K/Akt pathway, such as Sipeimine and Rosavidin, from Fritillariae Cirrhosae Bulbus and Rhodiola crenulate respectively (Huang, Jia, et al. 2023; Huang, Shen, et al. 2023). The gingerols, zingiberenes, gingerones and shogaols were characteristic ingredients of Zingiberaceae plants, and less in other plants. Interestingly, galangal, as another Zingiberaceae plants, also dislayed the markedly PI3K/Akt pathway inhibition (Wu et al. 2024), which also indicated that gingerols might be one of the main active components of JGAE against CA via modulating the cascade reaction.

## 5 Conclusions

This study demonstrated that the effect of JGAE against CA was related to modulating the imbalance of Th1/Th2, reducing the production of mucin, enhancing aquaporin expressions, and restoring acidic metabolites, while the involved mechanism was related to suppressing the excessive activation of the PI3K/Akt pathway. Furthermore, the gingerol compounds might be the material basis. These findings suggest that JG, as a commonly consumed food, exhibits substantial alleviation against CA disease, and highlight its potential as a dietary intervention for CA management.

## Author Contributions

H.D.: conceptualization, X.W. and H.Y.; data curation, X.S.; funding acquisition, H.Y., B.Y. and F.D.; investigation, Z.L.; methodology, H.D., F.D. and X.W.; software, X.W.; supervision, H.Y. and B.Y.; visualization, X.S. and F.D.; writing – original draft, X.S.; writing – review editing, B.Y.

## Ethics Statement

All animal experiments were approved by the Animal Ethics Committee of Nanjing University of Chinese Medicine (No. 202202A040, 202207A054 and 202302A058) and comply with the ARRIVE guidelines.

## Conflicts of Interest

The authors declare no conflicts of interest.

## Supporting information


**Data S1:** fsn371249‐sup‐0001‐Supinfo.docx.

## Data Availability

The original contributions presented in the study are included in the article, further inquiries can be directed to the corresponding author.
