# Supplementary material for: Jun Ginger Extract Improves Cold‐Induced Asthma by Inhibiting Airway Inflammation via PI3K/AKT Pathway
Source: Food Sci Nutr. 2025 Nov 19;13(11):e71249. doi: 10.1002/fsn3.71249 (PMC12629912; doi:10.1002/fsn3.71249)
Supplement: Supplementary file 1 — Data S1: fsn371249‐sup‐0001‐Supinfo.docx. [file FSN3-13-e71249-s001.docx]

***Supplementary Material***

Jun Ginger extract improves cold-induced asthma by inhibiting airway inflammation via PI3K/AKT pathway

Xueqing Wang ^1^, Xiaoxiong Song ^2^, Haokang Ding ^2^, Ziyan Liu ^2^, Fengjiao Deng ^2^, Hui Yan ^2,^*, Bin Yu ^1,^*

1 Jiangsu Collaborative Innovation Center of Chinese Medicinal Resources Industrialization, National and Local Collaborative Engineering Center of Chinese Medicinal Resources Industrialization and Formulae Innovative Medicine, Nanjing University of Chinese Medicine, Nanjing, 210023, China.

2 Jiangsu Key Laboratory of Efficacy and Safety Evaluation of Traditional Chinese Medicine, Nanjing University of Chinese Medicine, Nanjing, 210023, China.

* Correspondence: yanhui@njucm.edu.cn (H.Y.); yubin@njucm.edu.cn (B.Y.)

Xueqing Wang and Xiaoxiong Song contributed equally to this work

**E-mails:** wangxq393@163.com (X. Wang), songxiaoxiong6@163.com (X. Song), eliseoding@foxmail.com (H. Ding), 041321328@njucm.edu.cn (Z. Liu), 2197339183@qq.com (F. Deng), yanhui@njucm.edu.cn (H. Yan), yubin@njucm.edu.cn (B. Yu).

**Contents**

**Abbreviations**--------------------------------------------------------------------------------------------------------1

**Table S1.** The primer sequence used for qRT-PCR-------------------------------------------------------2

**Table S2.** Identified metabolites in metabolomic analysis-----------------------------------------------3

**Figure S1.** The classical difference between CDG and JG on the morphology of their cross-section-----------------------------------------------------------------------------------------------------------7

**Figure S2.** Establishment of the CA mice-------------------------------------------------------------------7

**Figure S3.** JGAE displayed the most significant efficacy in improving weight, airway hyperresponsiveness and pathological structural changes--------------------------------------------------8

**Figure S4.** JGAE displayed the best effect in improving inflammation in CA mice------------------9

**Figure S5.** Extract iron chromagraphy and MS/MS spectrum of JGAE in positive ion model----10

**Figure S6.** Extract iron chromagraphy and MS/MS spectrum of JGAE in negative ion model---10

**Figure S7.**  JGAE inhibited the inflammatory reaction of the CA mice-------------------------------11

**Figure S8.** The construction of the inflammation model of the BEAS-2B cells and the optimization of JGAE dosage-----------------------------------------------------------------------------------11

**Figure S9.** Metabolomics analysis result of JGAE against CA in mice lung tissues----------------12

**Abbreviations**

| **Abbreviations** | **Full names** |
| --- | --- |
| BALF | Bronchoalveolar lavage fluid |
| BEAS-2B | Normal human bronchial epithelial cells |
| CA | Cold-induced asthma |
| CDC | Conventional dried ginger |
| CDGAE | Aqueous-soluble extract of conventional dried ginger |
| CDGVOE | Volatile oil extract of conventional dried ginger |
| JG | Jun ginger |
| JGAE | Aqueous-soluble extract of Jun ginger |
| JGVOE | Volatile oil extract of Jun ginger |
| LGWWJX | Linggan Wuwei Jiangxin Prescription |
| LPS | Lipopolysaccharide |

**Table S1. The primer sequence used for qRT-PCR**

| Primer | Sequence |
| --- | --- |
| MUC5AC (Mouse) | Forward TCACTCTACCACTCCCTGCTTCTG  Reverse CCTGACAATCCTGGCTACACATCG |
| GAPDH (Mouse) | Forward GACTCCACTCACGGCAAATTCAAC  Reverse GACACCAGTAGACTCCACGACATAC |
| IL-4 (Human) | Forward ACCGAGTTGACCGTAACAGACATC  Reverse GTGTCCTTCTCATGGTGGCTGTAG |
| IL-5 (Human) | Forward AGCCAATGAGACTCTGAGGATTCC  Reverse TTGACTCTCCAGTGTGCCTATTCC |
| IL-13 (Human) | Forward GCAGCATGGTATGGAGCATCAAC  Reverse AGACCTTGTGCGGGCAGAATC |
| MUC5AC (Human) | Forward CCCGCCCACCTCTTCTACCC  Reverse TGACCACCACGAGCCCATCC |
| GAPDH (Human) | Forward ACCCACTCCTCCACCTTTGAC  Reverse TCCACCACCCTGTTGCTGTAG |

**Table S2. Identified metabolites in metabolomic analysis**

| **Name** | **Metabolite.ID** | **Formula** | **ppm** |
| --- | --- | --- | --- |
| L-Arginine | 0.665_175.11911 | C_6_H_14_N_4_O_2_ | 0.866 |
| L-Threonine | 0.682_118.05103 | C_4_H_9_NO_3_ | 0.510 |
| L-Aspartic acid | 0.692_134.04486 | C_4_H_7_NO_4_ | 0.576 |
| Cytidine | 1.086_242.07809 | C_9_H_13_N_3_O_5_ | 0.624 |
| Ophthalmic acid | 1.299_290.13479 | C_11_H_19_N_3_O_6_ | 0.460 |
| L-kynurenine | 2.535_209.09234 | C_10_H_12_N_2_O_3_ | 1.281 |
| Pantothenic acid | 3.103_220.11813 | C_9_H_17_NO_5_ | 0.824 |
| 2-Aminoheptanoate | 3.274_146.11768 | C_7_H_15_NO_2_ | 0.892 |
| Indolelactic acid | 4.868_206.08144 | C_11_H_11_NO_3_ | 1.340 |
| Indoleacetic acid | 5.24_176.07079 | C_10_H_9_NO_2_ | 1.037 |
| N-Acetyl-a-D-glucosamine  1-phosphate | 0.64_300.04881 | C_8_H_16_NO_9_P | -0.612 |
| Acetohydroxamic acid | 0.675_76.03938 | C_2_H_5_NO_2_ | 0.945 |
| Phosphorylcholine | 0.684_184.07348 | C_5_H_14_NO_4_P | 0.845 |
| DL-Alanine | 0.686_90.05507 | C_3_H_7_NO_2_ | 1.250 |
| methyl 1-isopropyl-1H-1,2,3-benzotriazole-5-carboxylate | 0.704_258.06417 | C_11_H_13_N_3_O_2_ | 0.173 |
| L-Ergothioneine | 0.735_230.09580 | C_9_H_15_N_3_O_2_S | 0.127 |
| DL-Arginine | 0.788_173.10439 | C_6_H_14_N_4_O_2_ | -0.033 |
| 2-hydroxy-4-(methylthio)butyric acid | 1.1_150.05852 | C_5_H_10_O_3_S | -1.200 |
| Trans-cinnamate | 2.756_131.04930 | C_9_H_8_O_2_ | -1.216 |
| 3-Indoxyl sulphate | 3.1_212.00219 | C_8_H_7_NO_4_S | -0.512 |
| 1,5-Naphthalenediamine | 3.363_159.09191 | C_10_H_10_N_2_ | 1.459 |
| Daidzein | 5.898_255.06532 | C_15_H_10_O_4_ | 0.523 |
| (15Z)-9,12,13-Trihydroxy-15-octadecenoic acid | 7.156_329.23330 | C_18_H_34_O_5_ | -0.136 |
| NP-016928 | 7.421_299.20071 | C_20_H_28_O_3_ | -0.570 |
| ent-prostaglandin E2 | 7.582_333.20706 | C_20_H_32_O_5_ | -0.511 |
| Prostaglandin A2 | 7.803_317.21116 | C_20_H_30_O_4_ | -0.539 |
| (4aS,9aR)-2-Benzoyl-7-[2-(1H-imidazol-4-yl)ethyl]decahydro-6H-pyrido[3,4-d]azepin-6-one | 8.854_367.21488 | C_21_H_26_N_4_O_2_ | 5.538 |
| NP-020521 | 8.862_279.23133 | C_18_H_32_O_3_ | -0.608 |
| (+/-)12(13)-Dihome | 8.917_295.22762 | C_18_H_34_O_4_ | -0.573 |
| L-Arginine | 0.665_175.11911 | C_6_H_14_N_4_O_2_ | 0.866 |
| **Name** | **Metabolite.ID** | **Formula** | **ppm** |
| L-Threonine | 0.682_118.05103 | C_4_H_9_NO_3_ | 0.510 |
| L-Aspartic acid | 0.692_134.04486 | C_4_H_7_NO_4_ | 0.576 |
| 20-Hydroxy-(5Z,8Z,11Z,14Z)-eicosatetraenoic acid | 8.984_319.22771 | C_20_H_32_O_3_ | -0.483 |
| 11(Z),14(Z),17(Z)-Eicosatrienoic acid | 9.206_307.26317 | C_20_H_34_O_2_ | 0.041 |
| Labdanolic acid | 9.362_323.25910 | C_20_H_36_O_3_ | -0.208 |
| Palmitoyl ethanolamide | 9.623_300.28978 | C_18_H_37_NO_2_ | 0.028 |
| 3-Hydroxybutyric acid | 0.718_103.04007 | C_4_H_8_O_3_ | -0.003 |
| Methyl 4-(aminomethyl)benzoate | 0.741_166.08615 | C_9_H_11_NO_2_ | 0.199 |
| Sitagliptin | 0.769_408.12594 | C_16_H_15_F_6_N_5_O | -3.788 |
| L-(-)-Methionine | 1.08_150.05850 | C_5_H_11_NO_2_S | 1.428 |
| Methyl 2-[(3S)-1-(tetrahydro-2H-pyran-4-yl)-3-pyrrolidinyl]-1,3-benzoxazole-7-carboxylate | 2.752_331.16553 | C_18_H_22_N_2_O_4_ | 1.137 |
| 4-(dimethylamino)benzaldehyde N-(4,5-dihydro-1H-imidazol-2-yl)hydrazone | 2.847_232.15459 | C_12_H_17_N_5_ | 0.889 |
| 4-Amino-2-(4-methylpiperazino)-5-pyrimidinecarbonitrile | 2.928_219.13416 | C_10_H_14_N_6_ | -0.468 |
| [3,5-di(tert-butyl)-1H-pyrazol-1-yl](4-methyl-1,2,3-thiadiazol-5-yl)methanone | 2.98_307.15863 | C_15_H_22_N_4_OS | -5.073 |
| NP-012017 | 3.257_276.18060 | C_13_H_22_O_5_ | -0.255 |
| [(3R,4S)-1-Acetyl-3-{2-[4-(4-fluorophenyl)-1-piperazinyl]ethyl}-4-piperidinyl]acetic acid | 3.343_414.21864 | C_21_H_30_FN_3_O_3_ | 0.660 |
| Indole | 3.363_118.06537 | C_8_H_7_N | 0.562 |
| Taurochenodeoxycholic acid | 7.457_482.29036 | C_26_H_45_NO_6_S | 2.092 |
| Norcholic acid | 7.903_412.30583 | C_23_H_38_O_5_ | -0.361 |
| N-{4-[(2R,3R)-3-(Hydroxymethyl)-4-isopropyl-5-oxo-2-morpholinyl]phenyl}-2-pyrazinecarboxamide | 8.861_353.16157 | C_19_H_22_N_4_O_4_ | 0.432 |
| Sparfloxacin | 9_375.16188 | C_19_H_22_F_2_N_4_O_3_ | -0.487 |
| N-[3,5-di(tert-butyl)-1-methyl-1H-pyrazol-4-yl]-5-methyl-1,2,3-thiadiazole-4-carboxamide | 9.531_336.18279 | C_16_H_25_N_5_OS | -0.459 |
| (S)-Lipoic acid | 0.625_207.05064 | C_8_H_14_O_2_S_2_ | -0.738 |
| Stipitatonate; | 0.633_209.00859 | C_9_H_4_O_6_ | -0.783 |
| Benzo[b]naphtho[2,1-d]thiophene; 1,2-Benzo-9-thiafluorene; | 0.64_235.05664 | C_16_H_10_S | 0.253 |
| (R)-dihydrolipoamide | 0.692_208.08166 | C_8_H_17_NOS_2_ | -0.408 |
| **Name** | **Metabolite.ID** | **Formula** | **ppm** |
| Glutamine hydroxamate | 0.73_145.06087 | C_5_H_10_N_2_O_4_ | -0.379 |
| Secnidazole | 0.743_186.08739 | C_7_H_11_N_3_O_3_ | -0.009 |
| 2-Amino-6-methyl-2,3,6,7-tetrahydro-1H-pteridin-4-one | 0.875_182.10384 | C_7_H_11_N_5_O | 0.375 |
| tetrahydrothiazine | 1.099_104.05305 | C_4_H_9_NS | 1.137 |
| Allo-hydroxycitric acid lactone | 1.44_191.01898 | C_6_H_6_O_7_ | 0.199 |
| 20-Hydroxy-(5Z,8Z,11Z,14Z)-eicosatetraenoic acid | 8.984_319.22771 | C_20_H_32_O_3_ | 0.185 |
| 11(Z),14(Z),17(Z)-Eicosatrienoic acid | 9.206_307.26317 | C_20_H_34_O_2_ | -0.483 |
| Labdanolic acid | 9.362_323.25910 | C_20_H_36_O_3_ | 0.041 |
| Palmitoyl ethanolamide | 9.623_300.28978 | C_18_H_37_NO_2_ | -0.208 |
| 3-Hydroxybutyric acid | 0.718_103.04007 | C_4_H_8_O_3_ | 0.028 |
| Methyl 4-(aminomethyl)benzoate | 0.741_166.08615 | C_9_H_11_NO_2_ | -0.003 |
| N1-[4-(trifluoromethoxy)phenyl]-2-[3-(1H-pyrrol-1-yl)-2-pyridyl]hydrazine-1-carboxamide | 0.768_378.11581 | C_17_H_14_F_3_N_5_O_2_ | -0.399 |
| Sitagliptin | 0.769_408.12594 | C_16_H_15_F_6_N_5_O | -0.379 |
| L-(-)-Methionine | 1.08_150.05850 | C_5_H_11_NO_2_S | 1.428 |
| Methyl 2-[(3S)-1-(tetrahydro-2H-pyran-4-yl)-3-pyrrolidinyl]-1,3-benzoxazole-7-carboxylate | 2.752_331.16553 | C_18_H_22_N_2_O_4_ | 1.137 |
| 4-(dimethylamino)benzaldehyde N-(4,5-dihydro-1H-imidazol-2-yl)hydrazone | 2.847_232.15459 | C_12_H_17_N_5_ | 0.889 |
| 4-Amino-2-(4-methylpiperazino)-5-pyrimidinecarbonitrile | 2.928_219.13416 | C_10_H_14_N_6_ | -0.468 |
| 7-Ketocholesterol | 10.061_401.34140 | C_27_H_44_O_2_ | -0.507 |
| PE(O-16:0/0:0) | 10.252_438.29884 | C_21_H_46_NO_6_P | -0.017 |
| 1-(7Z-Hexadecenoyl)-2-(4Z,7Z,10Z,13Z,16Z,19Z-docosahexaenoyl)-sn-glycero-3-phosphocholine | 10.943_804.55414 | C_46_H_78_NO_8_P | -0.353 |
| PE(17:1(9Z)/22:6(4Z,7Z,10Z,13Z,16Z,19Z)) | 10.969_776.52007 | C_44_H_74_NO_8_P | 0.543 |
| Calcium ionophore | 11.322_685.53717 | C_38_H_72_N_2_O_8_ | -0.311 |
| PC(22:5(4Z,7Z,10Z,13Z,16Z)/P-18:1(11Z)) | 11.819_818.60624 | C_48_H_84_NO_7_P | 0.150 |
| PC(18:4(6Z,9Z,12Z,15Z)/P-18:1(11Z)) | 11.877_764.55707 | C_44_H_78_NO_7_P | 0.516 |
| PC(18:2(9Z,12Z)/P-18:1(11Z)) | 11.904_768.59031 | C_44_H_82_NO_7_P | -0.236 |
| PC(16:0/P-18:1(11Z)) | 12.019_744.59071 | C_42_H_82_NO_7_P | 0.236 |
| SM(d18:1/18:0) | 12.046_731.60660 | C_41_H_83_N_2_O_6_P | 0.621 |
| PC(18:1(11Z)/P-18:1(11Z)) | 12.087_770.60585 | C_44_H_84_NO_7_P | 0.613 |
| sufac#1 | 12.11_339.18276 | C_15_H_30_O_6_S | 0.042 |
| **Name** | **Metabolite.ID** | **Formula** | **ppm** |
| PE(O-16:0/22:5(4Z,7Z,10Z,13Z,16Z)) | 12.395_752.55873 | C_43_H_78_NO_7_P | -0.245 |
| (4R,5R)-4,5-Dihydroxycyclohexa-1(6),2-diene-1-carboxylate; (3R,4R)-3,4-Dihydroxycyclohexa-1,5-diene-1-carboxylate | 3.264_157.04962 | C_7_H_8_O_4_ | -0.097 |
| 11-amino-undecanoic acid | 6.281_202.18038 | C_11_H_23_NO_2_ | 0.553 |
| 3-methoxy-2-(3-methylbut-2-enyl)-5-pentylphenol | 6.418_280.22713 | C_17_H_26_O_2_ | 1.098 |
| 4-(2-((4-(2-(Pyridin-2-yl)-5,6-dihydro-4H-pyrrolo[1,2-b]pyrazol-3-yl)quinolin-7-yl)oxy)ethyl)morpholine | 6.464_442.22589 | C_26_H_27_N_5_O_2_ | 0.649 |
| Lipoxin C4 | 7.081_642.30560 | C_30_H_47_N_3_O_10_S | 0.485 |
| 5-(4-acetyloxy-3-hydroxy-2,5,5,8a-tetramethyl-3,4,4a,6,7,8-hexahydronaphthalen-1-yl)-3-methylpentanoic acid | 7.769_379.24908 | C_22_H_36_O_5_ | 0.174 |
| Bacillamidin A | 7.851_330.22754 | C_17_H_31_NO_5_ | 0.229 |
| 19,20-DiHDPA | 7.931_363.25302 | C_22_H_34_O_4_ | 0.000 |
| PGF2a ethanolamide | 7.936_398.29013 | C_22_H_39_NO_5_ | 0.020 |
| (2Z)-2-[(E)-6-(hydroxymethyl)-2,4,8,10-tetramethyldodec-2-enylidene]-4-methylpentanedioic acid | 8.198_414.32166 | C_23_H_40_O_5_ | 0.078 |
| (+)-Lysergic acid | 8.306_267.11360 | C_16_H_16_N_2_O_2_ | 0.430 |
| 3-Hydroxy-11Z-octadecenoylcarnitine | 8.577_442.35289 | C_25_H_47_NO_5_ | -1.134 |
| Arachidoyl Ethanolamide | 8.66_356.35218 | C_22_H_45_NO_2_ | 0.436 |
| 5-oxo-6E,8Z-tetradecadienoic acid | 8.821_239.16425 | C_14_H_22_O_3_ | -0.366 |
| Gonal | 8.857_261.22055 | C_18_H_28_O | 0.313 |
| Proglumide | 8.861_335.19770 | C_18_H_26_N_2_O_4_ | -0.285 |
| N-(1,3-Dihydroxyoctadec-4-en-2-yl)acetamide | 8.861_342.29951 | C_20_H_39_NO_3_ | 0.350 |
| Propargite | 8.862_333.15059 | C_19_H_26_O_4_S | -0.224 |
| Entinostat | 8.911_377.16203 | C_21_H_20_N_4_O_3_ | -0.375 |
| 5alpha-Androstane-3beta,7alpha,17beta-triol | 8.969_307.22775 | C_19_H_32_O_3_ | 0.323 |
| Benzene, p-di-tert-butoxy- | 9_223.16956 | C_14_H_22_O_2_ | -0.386 |
| LysoPE(22:6(4Z,7Z,10Z,13Z,16Z,19Z)/0:0) | 9.243_526.29356 | C_27_H_44_NO_7_ P | 1.352 |
| Stigmatellin Y | 9.314_502.31694 | C_29_H_40_O_6_ | 1.405 |
| LysoPC(22:4(7Z,10Z,13Z,16Z)/0:0) | 9.531_572.37118 | C_30_H_54_NO_7_P | 1.220 |
| LysoPC(P-18:0/0:0) | 9.762_508.37652 | C_26_H_54_NO_6_P | 0.180 |


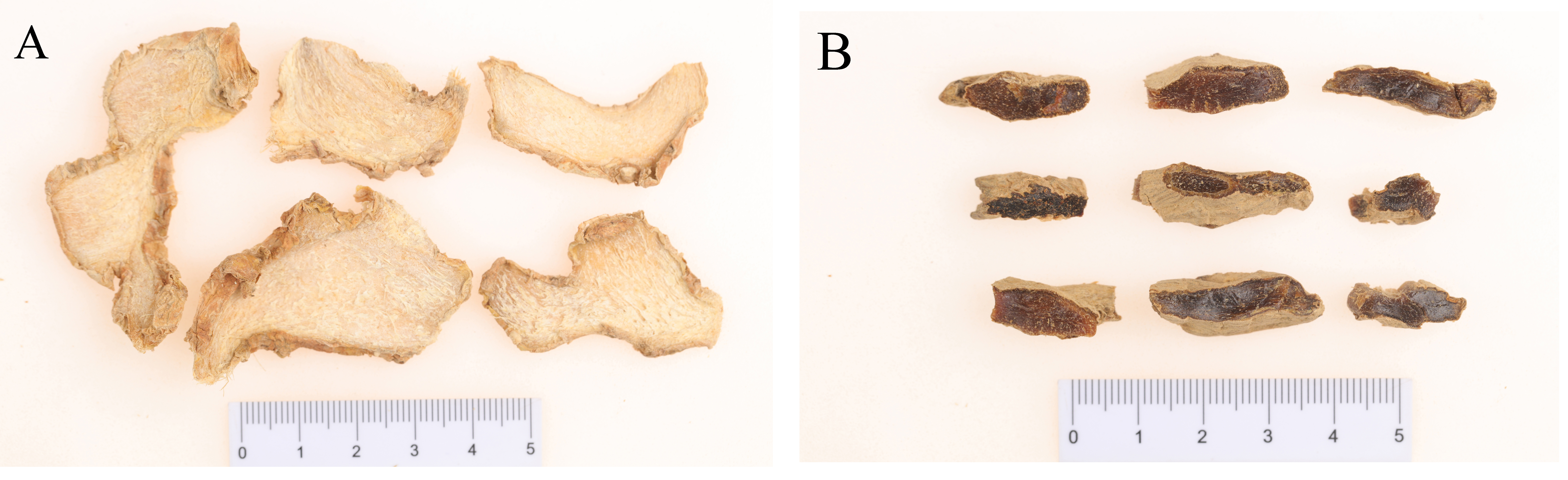


**Figure S1.** The classical difference between CDG and JG on the morphology of their cross-section. (A) The cross-section of CDG is pale yellow or light brown with fibrous structures inside. (B) The cross-section of JG is dark brown or black with a glassy texture. CDG: conventional dried ginger; JG: Jun ginger.


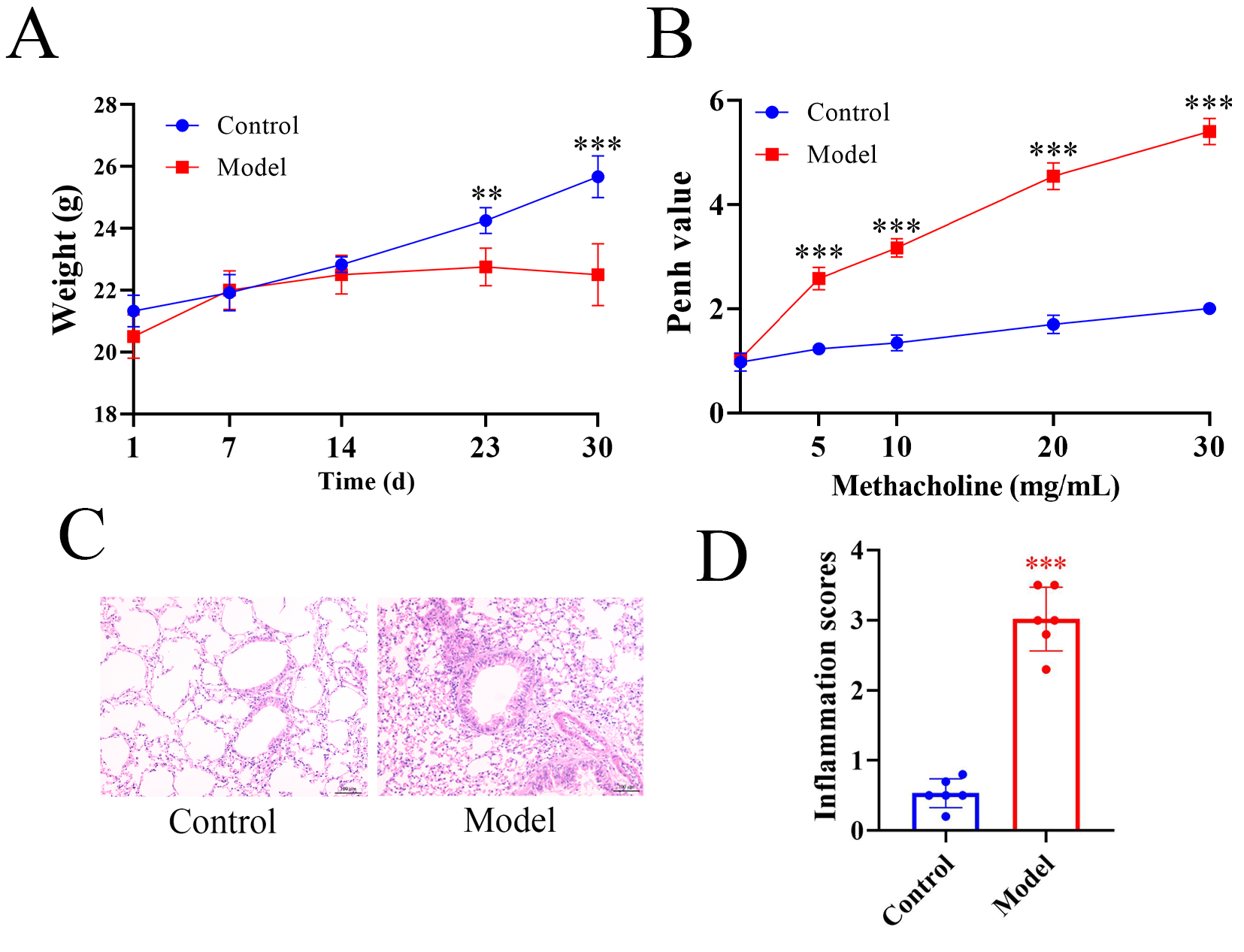


**Figure S2.** Establishment of CA mice. (A) Time-dependent line chart of body weight during the construction of the CA mice (n = 6). (B) Time-dependent line chart of Penh value during the construction of the CA mice (n = 6). (C-D) Pathological images and inflammatory scores of lung tissues of the CA mice (n=6, ×200). *^**^ p*<0.01, *^***^ p*<0.001 *vs.* the control group.

**
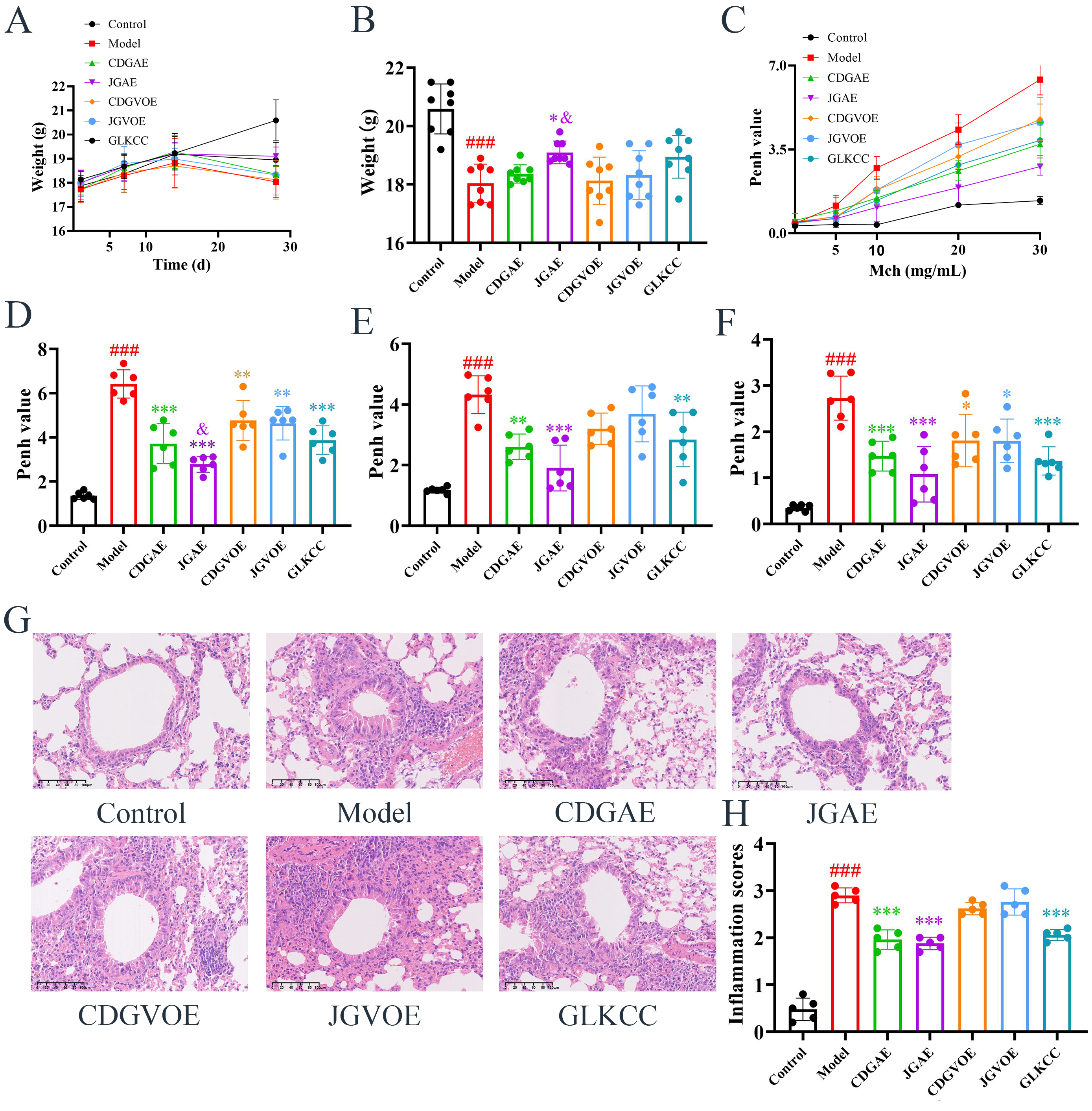
**

**Figure S3.** JGAE displayed the most significant efficacy in improving weight, airway hyperresponsiveness and pathological structural changes. (A) The body weights of the mice throughout the experiment (n = 8). (B) The body weights of the mice on day 28 in each group (n = 8). (C) The changing trends of the Penh value of the mice along with the increase of the Mech concentration (n = 6). (D-F) The Penh values of the mice stimulated by 30, 20 and 10 mg/mL of Mech, respectively (n = 6). (G-H) Pathological changes and inflammatory scores of lung tissues in the different groups (n=5, ×200). *^###^ p*<0.001 *vs.* the control group; *^*^ p*<0.05, *^**^ p*<0.01, *^***^ p*<0.001 *vs.* the model group; ^&^ *p*<0.05, ^&&^ *p*<0.01 *vs.* the CDGAE group. CDGAE: aqueous-soluble extract of conventional dried ginger; CDGVOE: volatile oil extract of conventional dried ginger; JGAE: aqueous-soluble extract of Jun ginger; JGVOE: volatile oil extract of Jun ginger; LGWWJX: Linggan Wuwei Jiangxin Prescription.

**
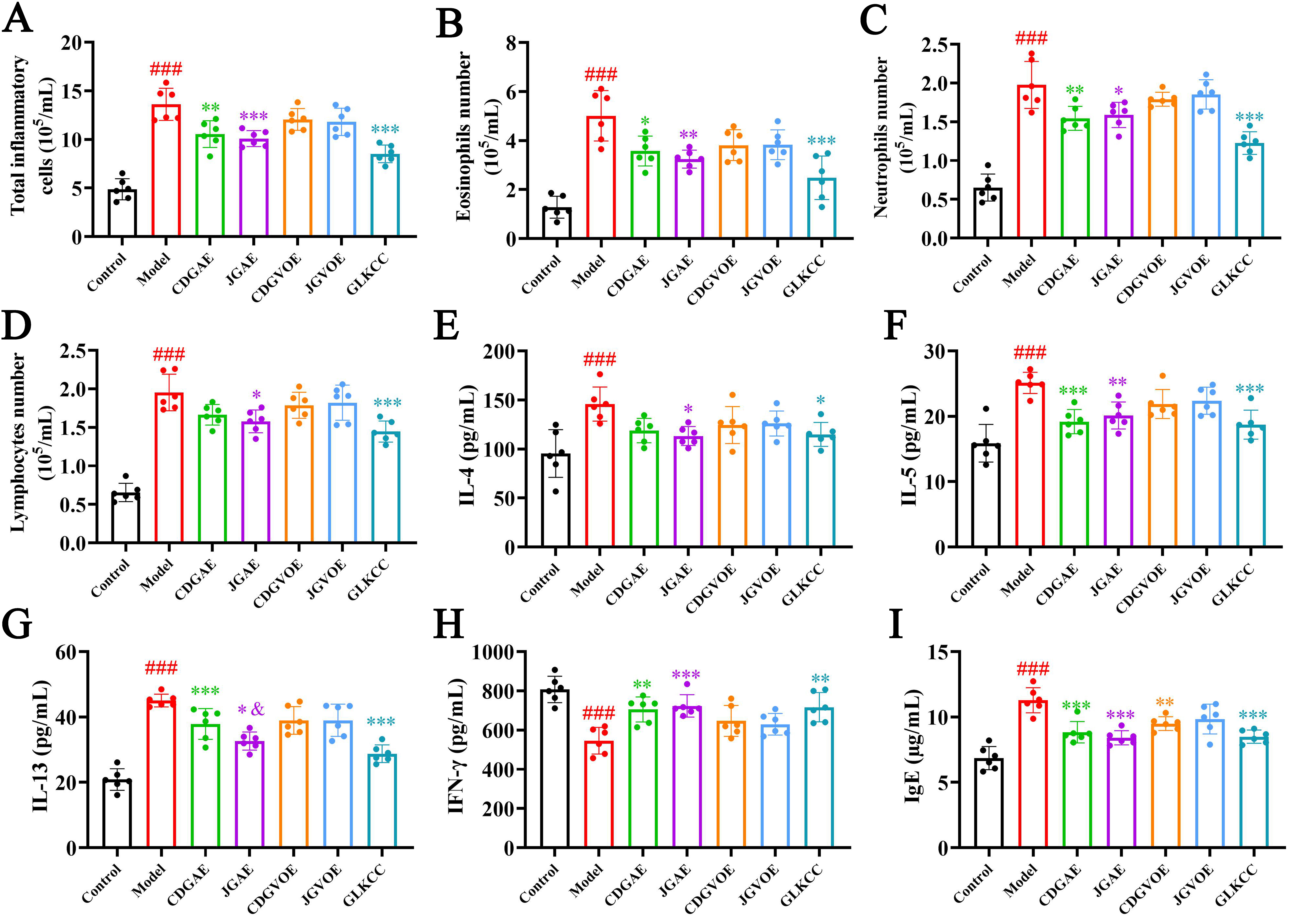
**

**Figure S4.** JGAE displayed the best effect in improving inflammation in CA mice (n = 6). (A-D) The total number of inflammatory cells, eosinophils, neutrophils and lymphocytes in the BALF of mice, respectively. (E-G) The concentrations of IL-4, IL-5 and IL-13 in the BALF of mice, respectively. (H-I) The concentrations of IFN-γ and IgE in the serum of mice. *^###^ p*<0.001 *vs.* the control group; *^*^ p*<0.05, *^**^ p*<0.01, *^***^ p*<0.001 *vs.* the model group; ^&^ *p*<0.05 *vs.* the CDGAE group.

**
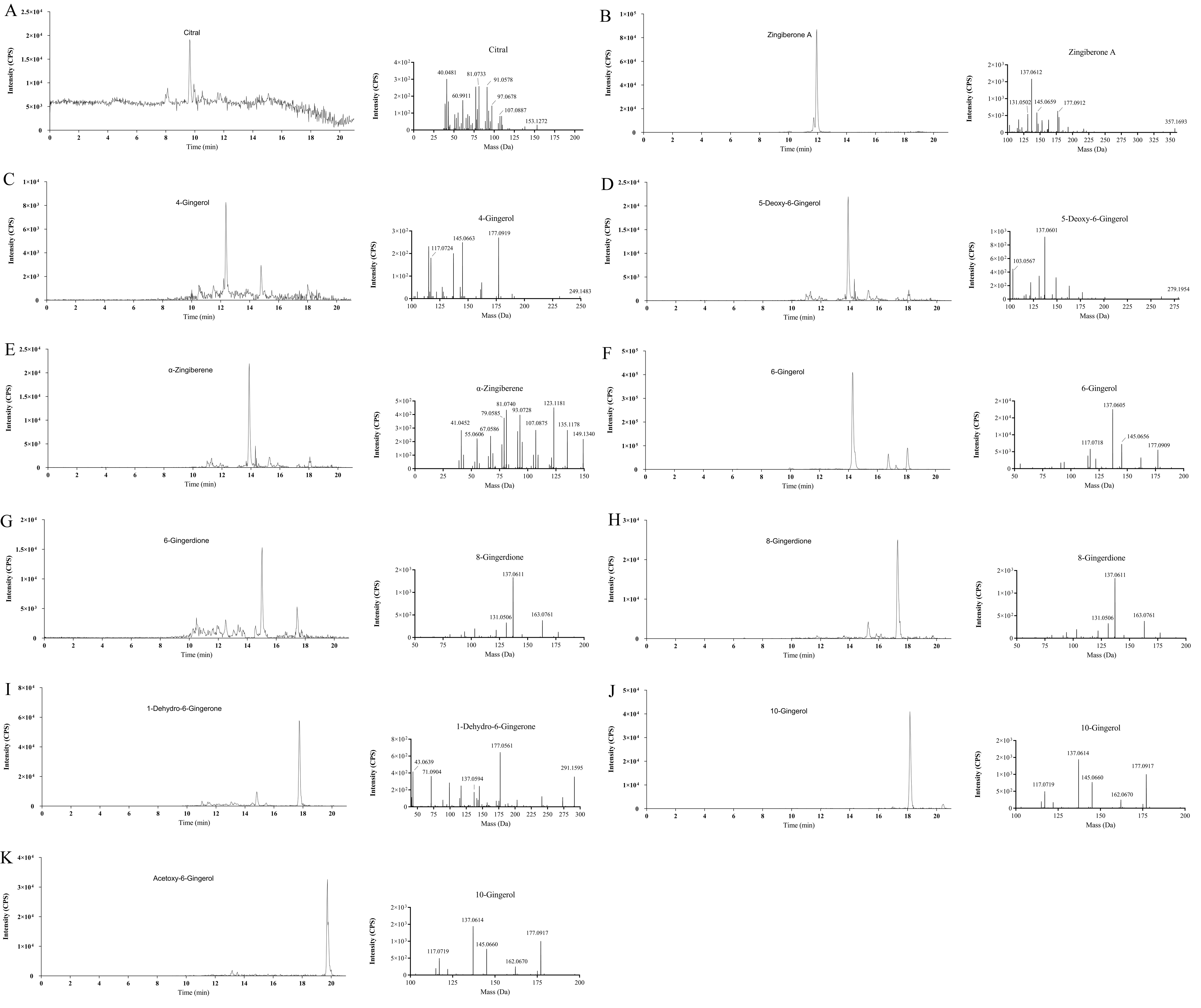
**

**Figure S5.** Extract iron chromagraphy and MS/MS spectrum of JGAE in positive ion model. A~K were Citral, Zingiberone A, 4-Gingerol, 5-Deoxy-6-Gingerol, α-Zingiberene, 6-Gingerol, 6-Gingerdione, 8-Gingerdione, 1-Dehydro-6-Gingerone, 10-Gingerol and Acetoxy-6-Gingerol, respectively.

**
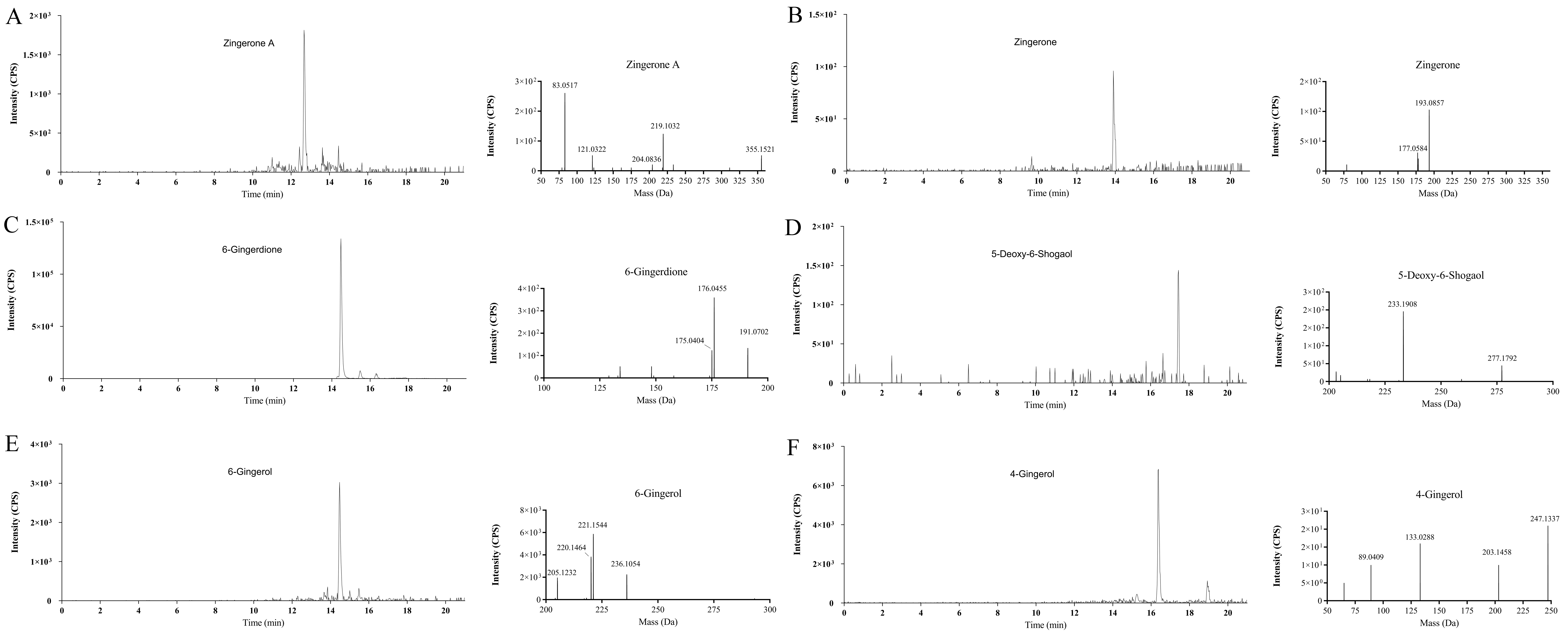
**

**Figure S6.**  Extract iron chromagraphy and MS/MS spectrum of JGAE in negative ion model. A~F were Zingerone A, Zingerone, 6-Gingerdione, 5-Deoxy-6-Shogaol, 6-Gingerol and 4-Gingerol, respectively.





**Figure S7.** JGAE inhibited the inflammatory reaction of the CA mice (n = 6). (A-D) The total number of inflammatory cells, eosinophils, neutrophils and lymphocytes in the BALF of mice, respectively. (E-G) The concentrations of IL-4, IL-5 and IL-13 in the BALF of mice, respectively. (H-I) The concentrations of IFN-γ and IgE in the serum of mice. *^###^ p*<0.001 *vs.* the control group; *^*^ p*<0.05, *^**^ p*<0.01, *^***^ p*<0.001 *vs.* the model group.


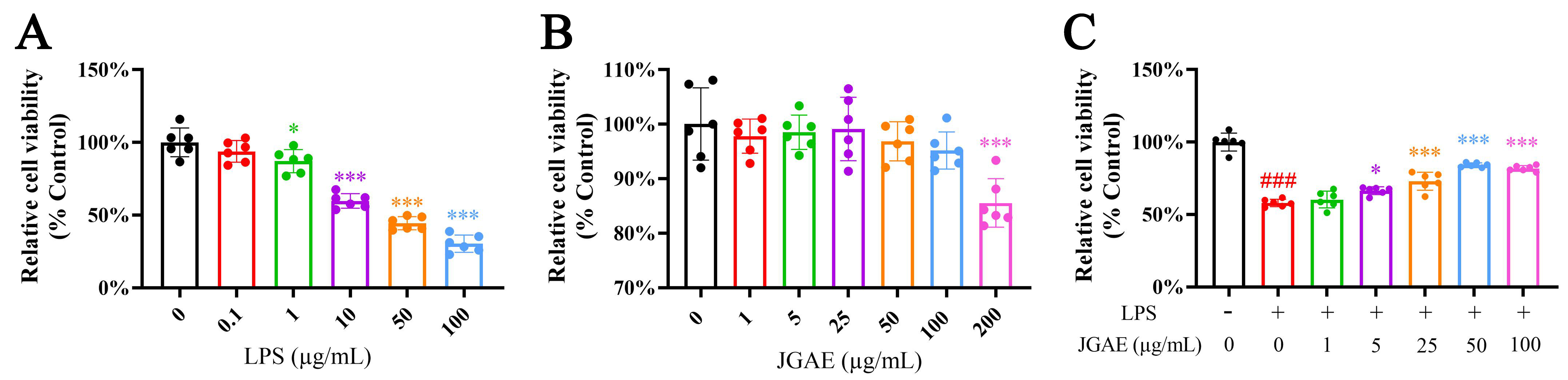


**Figure S8.** The construction of the inflammation model of the BEAS-2B cells and the optimization of JGAE dosage (n = 6). (A) Effects of LPS with various concentrations on the viability of BEAS-2B cells. *^*^ p*<0.05, *^***^ p*<0.001 *vs.* the 0 µg/mL group. (B) Effects of JGAE with various concentrations on the viability of normal BEAS-2B cells. *^***^* *p*<0.001 *vs.* 0 µg/mL group. (C) Effects of JGAE with various concentrations on the viability of BEAS-2B cells injured by LPS. *^###^ p*<0.001 *vs.* the control group; *^*^ p*<0.05, *^***^ p*<0.001 *vs.* the model group.


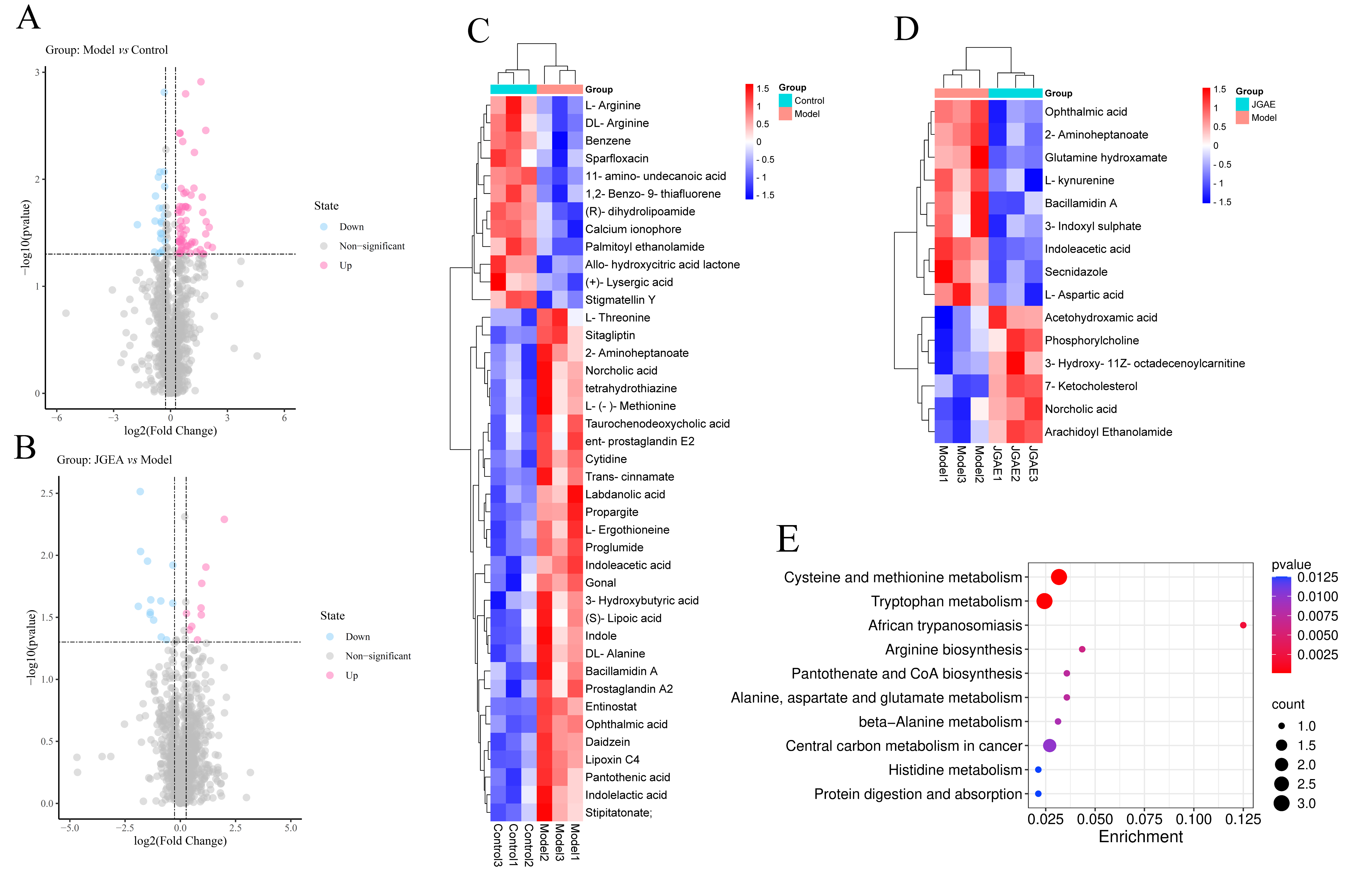


**Figure S9.** Metabolomics analysis result of JGAE against CA in the mice lung tissues (n=3). (A) The volcano plot of the differential metabolites in the lung tissue between the control group and the model group. (B) The volcano plot of the differential metabolites in the lung tissue between the model group and the JGAE (3.6 g/kg) group. (C) Heat map of differential metabolites in lung tissues between the control group and the model group. (D) Heat map of differential metabolites in lung tissues between the model group and the JGAE (3.6 g/kg) group. (E) KEGG enrichment result of differential metabolites between the model group and the JGAE (3.6 g/kg) group.
